# Adverse perinatal outcomes associated with different classes of antiretroviral drugs in pregnant women with HIV

**DOI:** 10.1097/QAD.0000000000004032

**Published:** 2024-10-15

**Authors:** Molly Hey, Lucy Thompson, Clara Portwood, Harriet Sexton, Mary Kumarendran, Zoe Brandon, Shona Kirtley, Joris Hemelaar

**Affiliations:** a National Perinatal Epidemiology Unit, Infectious Disease Epidemiology Unit, Nuffield Department of Population Health; b Centre for Statistics in Medicine, Nuffield Department of Orthopaedics, Rheumatology and Musculoskeletal Sciences, University of Oxford, Oxford, UK.

**Keywords:** antiretroviral therapy, HIV, low birthweight, neonatal death, pregnancy, preterm birth, small for gestational age

## Abstract

**Objective:**

Women with HIV (WHIV) are at an increased risk of adverse perinatal outcomes compared to women without HIV, despite antiretroviral therapy (ART). There is evidence that the risk of adverse perinatal outcomes may differ according to ART regimen. We aimed to assess the risk of adverse perinatal outcomes among WHIV receiving different classes of ART, compared to women without HIV.

**Design:**

A systematic review and meta-analysis

**Methods:**

We searched Medline, CINAHL, Global Health, and EMBASE for studies published between January 1, 1980, and July 14, 2023. We included studies which assessed the risk of 11 predefined adverse perinatal outcomes among WHIV receiving nonnucleoside reverse transcriptase inhibitor (NNRTI)-based ART, protease inhibitor based ART or integrase strand transfer inhibitor (INSTI)-based ART, compared to women without HIV. The perinatal outcomes assessed were preterm birth (PTB), very PTB (VPTB), spontaneous PTB (sPTB), low birthweight (LBW), very LBW (VLBW), term LBW, preterm LBW, small for gestational age (SGA), very SGA (VSGA), stillbirth and neonatal death (NND). Random effects meta-analyses examined the risk of each adverse outcome in WHIV receiving NNRTI-based, protease inhibitor based, or INSTI-based ART, compared with women without HIV. Subgroup and sensitivity analyses were conducted based on country income status, study quality, and timing of ART initiation. The protocol is registered with PROSPERO, CRD42021248987.

**Results:**

Of 108 720 identified citations, 22 cohort studies including 191 857 women were eligible for analysis. We found that WHIV receiving NNRTI-based ART (mainly efavirenz or nevirapine) are at an increased risk of PTB (risk ratio 1.40, 95% confidence interval 1.27–1.56), VPTB (1.94, 1.25–3.01), LBW (1.63, 1.30–2.04), SGA (1.53, 1.17–1.99), and VSGA (1.48, 1.16–1.87), compared with women without HIV. WHIV receiving protease inhibitor based ART (mainly lopinavir/ritonavir or unspecified) are at an increased risk of PTB (1.88, 1.55–2.28), VPTB (2.06, 1.01–4.18), sPTB (16.96, 1.01–284.08), LBW (2.90, 2.41–3.50), VLBW (4.35, 2.67–7.09), and VSGA (2.37, 1.84–3.05), compared with women without HIV. WHIV receiving INSTI-based ART (mainly dolutegravir) are at an increased risk of PTB (1.17, 1.06–1.30) and SGA (1.20, 1.08–1.33), compared with women without HIV.

**Conclusion:**

The risks of adverse perinatal outcomes are higher among WHIV receiving ART compared with women without HIV, irrespective of the class of ART drugs. This underlines the need to further optimize ART in pregnancy and improve perinatal outcomes of WHIV.

## Introduction

Each year, there are an estimated 1.3 million pregnant women with HIV (WHIV), 82% of whom receive antiretroviral therapy (ART) [1]. 90% of pregnant WHIV reside in sub-Saharan Africa, which has the highest rates of child mortality worldwide [2,3]. The United Nations’ Sustainable Development Goal 3 target 3.2 aspires to decrease neonatal and under-5 mortality to 12 and 25 per 1000 live births, respectively, by 2030 [4]. However, the majority of countries in sub-Saharan Africa are not on track to achieve these goals [2]. Preterm birth (PTB) is the leading cause of neonatal and child mortality and morbidity globally [5], while babies born small for gestational age (SGA) account for 21.9% of neonatal deaths (NNDs) in low and middle-income countries (LMICs) [6]. In 2020, 14.6% of livebirths worldwide had low birthweight (LBW) [7]. Therefore, there is an urgent unmet need to improve perinatal outcomes in settings where HIV infection and neonatal and child mortality are most prevalent.

WHIV who do not receive ART have an increased risk of PTB, LBW, SGA, and stillbirth, especially in sub-Saharan Africa [8]. The WHO recommends that all pregnant WHIV receive ART to improve maternal health and reduce vertical HIV transmission [9]. However, pregnant WHIV who receive ART remain at an increased risk of PTB, LBW, term LBW, SGA, and very SGA (VSGA) compared to women without HIV [10]. In the past three decades, an estimated 2 million preterm, LBW, and SGA newborns in sub-Saharan Africa have been attributed to maternal HIV infection and ART [11].

ART consists of a backbone of two nucleoside reverse transcriptase inhibitors (NRTIs), combined with a “third drug,” which may be an integrase strand transfer inhibitor (INSTI), nonnucleoside reverse transcriptase inhibitor (NNRTI), protease inhibitor, or NRTI. Currently, the WHO recommends INSTI dolutegravir (DTG)-based ART as the preferred first-line ART regimen in adults and pregnant women, NNRTI efavirenz (EFV)-based ART as an alternative first line, and protease inhibitor based ART as second-line if first-line regimens fail [9]. However, the existence of differential risks of adverse perinatal outcomes associated with different ART regimens is uncertain, with conflicting data reported [12–17]. Recent RCTs of ART regimens initiated during pregnancy showed no differences in composite perinatal outcomes between DTG-based ART and EFV-based ART, although there was an increase in NND associated with EFV-based ART [13,14]. A further RCT showed that WHIV receiving INSTI raltegravir-based ART had similar adverse perinatal outcomes as WHIV receiving EFV-based ART [15]. Meanwhile, meta-analyses of cohort studies comparing different ART classes found an association of protease inhibitor based ART with an increased risk of SGA and VSGA compared to NNRTI-based ART [16,17].

On the basis of the available evidence, it is unclear whether any ART regimen received by WHIV reduces their risk of adverse perinatal outcomes to the level of women without HIV. To fill this evidence gap, we conducted a systematic review and meta-analysis to assess the risk of a broad range of important adverse perinatal outcomes among WHIV receiving INSTI-based ART, NNRTI-based ART and protease inhibitor based ART, compared to women without HIV.

## Materials and methods

### Search strategy

This systematic review and meta-analysis was conducted in concordance with the Cochrane guidelines. A comprehensive search strategy was devised by a specialist librarian (S.K.) and used with four electronic databases [Medline, CINAHL (Ebscohost), Global Health (Ovid), EMBASE (Ovid)] to identify studies published between January 1, 1980, and July 14, 2023 (Appendix 1). Full texts and abstracts were reviewed, with no restrictions on study design, language or country. Citations were imported into EndNote reference manager (EndNote 21; Clarivate Analytics, Philadelphia, Pennsylvania, USA) and deduplicated.

### Study selection and eligibility criteria

Studies containing data on the occurrence of predefined adverse perinatal outcomes among WHIV receiving ART and women without HIV were eligible. Titles and abstracts of retrieved citations were reviewed, and full text manuscripts were assessed against the eligibility criteria by at least two independent investigators (M.H., L.T., C.P., H.S., M.K., and Z.B.). Inclusion criteria were study design (cohort studies), population (pregnant women), exposure (WHIV receiving NNRTI-based, protease inhibitor based or INSTI-based ART), comparator (women without HIV), and perinatal outcomes defined as follows: PTB (birth <37^+0^ weeks gestation) [5]; very PTB (VPTB, birth <32^+0^ weeks gestation) [5]; spontaneous PTB (sPTB, spontaneous birth <37^+0^ weeks gestation); LBW (<2500 g) [7]; very LBW (VLBW, <1500 g); SGA (birthweight for gestational age <10^th^ centile) [6] or VSGA (birthweight for gestational age <3^rd^ centile) according to the reference chart used at the study site; stillbirth (newborn without any signs of life with birthweight at least 1000 g or gestational age ≥24^+0^ weeks gestation weeks or body length ≥35 cm); and NND (death of an infant in the first 28 days of life) [8]. Term and preterm LBW were defined according to the definitions for PTB and LBW. Perinatal outcome data were not included if outcomes were undefined or not defined according to our definitions. ART exposure was defined as receiving any combination of at least three antiretroviral drugs, categorized as NNRTI-based, protease inhibitor based, or INSTI-based ART, depending on the class of the “third drug.” Any ambiguities were resolved with the senior investigator (J.H.).

### Data extraction

At least two independent investigators (M.H., L.T., C.P., H.S., M.K., and Z.B.) extracted data on study and population characteristics. Data on class of ART exposure (as well as specific drugs), timing of ART initiation (preconception, antenatal, or mixed), and frequencies of adverse perinatal outcomes among WHIV receiving different classes of ART and women without HIV were extracted. Unadjusted and adjusted relative risks and 95% confidence intervals (95% CIs) of adverse perinatal outcomes of WHIV receiving different classes of ART, compared to women without HIV, were also extracted. All data were reviewed by the senior investigator (J.H.).

### Quality assessment

The quality of each study was assessed by at least two investigators (M.H., L.T., C.P., H.S., M.K., and Z.B.) and reviewed by the senior investigator (J.H.) using an adapted Newcastle–Ottawa Scale (Appendix 2). Studies were defined as “good,” “average,” or “poor” quality according to nine predefined criteria (Appendix 2).

### Statistical analysis

Outcome frequencies were used to calculate risk ratios and corresponding 95% CIs to assess the risk of adverse perinatal outcomes among WHIV receiving NNRTI-based, protease inhibitor based, or INSTI-based ART, compared with women without HIV in individual studies. If two or more studies reported data for the same exposure comparison and outcome, a random effects meta-analysis was conducted. Meta-analyses were represented in forest plots and the *I*^2^ statistic used to quantify heterogeneity due to clinical and methodological variability between studies. Funnel plots were used to assess small study effects and the Peters’ test was used in meta-analyses containing more than 10 studies (Appendix 6) [18]. Subgroup analyses were performed to assess the role of specific NNRTI drugs [i.e., efavirenz (EFV) and nevirapine (NVP)], timing of ART initiation (preconception and antenatal), country income status (high or low and middle-income), and study quality (good, average or poor) (Appendix 4). A sensitivity analysis was performed to investigate whether adjustment for confounders impacted the associations between exposures and perinatal outcomes (Appendix 5). Statistical analyses were done with StataBE version 18 (College Station, Texas, USA). The review is reported according to Preferred Reporting Items for Systematic Reviews and Meta-Analyses (PRISMA) guidelines [19].

## Results

**Fig. 1:**
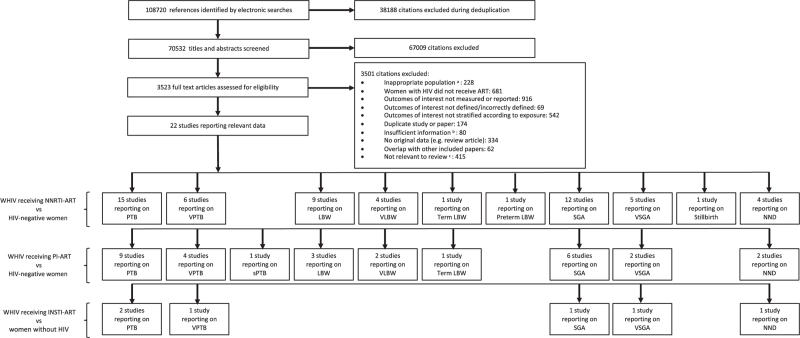
Study selection.

ART, antiretroviral therapy (triple drug therapy); INSTI, integrase strand transfer inhibitor; LBW, low birthweight; NND, neonatal death; NNRTI, nonnucleoside reverse transcriptase inhibitor; PI, protease inhibitor; PTB, preterm birth; SGA, small for gestational age; sPTB, spontaneous preterm birth; VLBW, very low birthweight; VPTB, very preterm birth; VSGA, very small for gestational age; WHIV, women with HIV. ^a^For example, women with HIV were not pregnant. ^b^For example, study did not provide relevant outcome data. ^c^For example, assisted reproductive technology.

Our search yielded 108 720 citations, of which 22 studies were included in our meta-analysis [20–41]. The number of studies reporting each perinatal outcome for WHIV receiving NNRTI-based ART, protease inhibitor based ART, or INSTI-based ART, compared to women without HIV, are shown in Fig. 1.

Table 1 summarizes the characteristics of each study. In total, eight prospective (36%) and 14 retrospective (64%) cohort studies reported data from 191 857 women across 10 countries. Fifteen studies (68%) including 175 839 women (92%) took place in LMICs, whereas seven studies (32%) with 16 018 women (8%) were conducted in high-income countries (HICs). Seven studies (32%) were classified as poor quality, 14 (64%) as average quality, and one (4%) as good quality (Table 1 and Appendix 2). Eighteen studies (82%) used methods to assess for potential confounders, including regression analysis, risk factor analysis, and matching. Of the 31 analyses reported by individual studies that adjusted for confounders, only five resulted in a change in the significance of the effect estimate (Appendix 5).

**Table 1:** Characteristics of studies.

Study	Country	Country income status	Cohort study design	Recruitment period	Number of women analysed	Population characteristics^a^	Method to correct for confounders	Method to estimate gestational age	Quality Assessment
Azria *et al.*[19]	France	High	Retrospective	1/2003 to 6/2007	300	Twins excluded, women recruited from a level III maternity unit, urban setting, hospital deliveries, 4.3% smoking during pregnancy, 1.7% history of IDU	Risk factor analysis, matching	First day of LNMP, corrected if needed by routine first trimester ultrasound	Average
Balogun *et al.*[20]	Canada	High	Prospective	9/2010 to 12/2015	104	Twins excluded, women recruited from 4 sites in Toronto, 0% smoking	Risk factor analysis, matching	LNMP confirmed by ultrasound (unspecified)	Average
Bengtson *et al.*[21]	South Africa	Middle	Prospective	3/2013 to 8/2015	1116	Twins excluded, women recruited from antenatal care clinics in Gugulethu Cape Town, urban setting, 17.2% alcohol use	None	Ultrasound (unspecified), LNMP, or symphysis-fundal height	Poor
Boer *et al.*[22]	Netherlands	High	Retrospective	12/1997 to 7/2003	294	First born twin included, women recruited from an academic medical centre, 12.9% smoking, 1.7% history of IDU	Regression analysis, matching	LNMP confirmed by first trimester ultrasound	Poor
Carceller *et al.*[23]	Canada	High	Retrospective	1997 to 2005	412	Recruited from a tertiary hospital in Montreal, urban setting, hospital deliveries	None	No description	Poor
Chen *et al.*[24]	Botswana	Middle	Retrospective	1/5/2009 to 30/4/2011	33148	First born twin included, hospital deliveries, 5.3% alcohol use, 1.7% smoking	Regression analysis, risk factor analysis	LNMP, symphysis-fundal height, or ultrasound (unspecified)	Average
Dadabhai *et al.*[25]	Malawi	Low	Prospective	1/2016 to 9/2017	1299	Twins excluded, 96% of deliveries occurred in healthcare facilities, urban setting	Regression analysis	Ballard score and LNMP	Average
Gagnon *et al.*[26]	Canada	High	Retrospective	1/1/2007 to 31/12/2012	384	Twins excluded, women recruited from tertiary referral centre, urban setting, all hospital deliveries, 5% smoking, 1% alcohol use, 2% IDU.	Regression, risk factor analysis	First trimester ultrasound or conception date by assisted reproduction if available	Average
Malaba *et al.*[27]	South Africa	Middle	Prospective	4/2013 to 8/2015	1793	Twins excluded, recruited from large community primary care facility, urban setting	Regression analysis, risk factor analysis	LNMP and symphysis-fundal height	Average
Malaba *et al.*[28]	South Africa	Middle	Prospective	4/2014 to 10/2016	1787	Twins excluded, women recruited from a large primary care antenatal clinic, urban setting	Regression analysis	LNMP and symphysis-fundal height	Average
Mehta *et al.*[29]	South Africa	Middle	Retrospective	7/10/2013 to 6/10/2014	10293	Twins included, women recruited from hospital, urban setting, hospital deliveries, 0.09% smoking, 0.2% alcohol use, 0.04% IDU	Risk factor analysis	LNMP, ultrasound (unspecified)	Average
Moodley *et al.*[30]	South Africa	Middle	Retrospective	7/2011 to 12/2011, 1/2014 to 6/2014	9847	Twins excluded, data abstracted from maternity registers of a regional hospital	Regression analysis, risk factor analysis	LNMP and/or ultrasound (unspecified)	Average
Olagbuji *et al.*[31]	Nigeria	Middle	Prospective	1/2007 to 12/2008	406	Twins excluded, women recruited from a tertiary referral centre, all delivered in a healthcare facility	Risk factor analysis	No description	Poor
Ramokolo *et al.*[32]	South Africa	Middle	Retrospective	10/2012 to 5/2013	8778	Women recruited from primary health facilities	Risk factor analysis	LNMP	Average
Rempis *et al.*[33]	Uganda	Low	Retrospective	2/2013 to 12/2013	412	Twins excluded, all deliveries in a private referral hospital	Risk factor analysis	No description	Poor
Santosa *et al.*[34]	South Africa	Middle	Prospective	28/5/2013 to 20/7/2016	633	Twins excluded, women recruited from hospital, 98.7% hospital deliveries, urban setting, 6.4% smoking, 8.2% alcohol	Regression analysis, risk factor analysis	Ultrasound <14 weeks	Good
Saums *et al.*[35]	United States of America	High	Retrospective	2011--2018	3729	Women recruited from hospital, urban setting, hospital deliveries, 11.5% smoking, 2.9% alcohol use, 13.4% IDU	Risk factor analysis	No description	Average
Sebitloane *et al.*[36]	South Africa	Middle	Retrospective	1/4/2011 to 30/4/2014	1461	Twins excluded, women recruited at a regional hospital, urban setting, hospital deliveries	None	No description	Poor
Snijdewind *et al.*[37]	Netherlands	High	Retrospective	1/1997 to 2/2015	10795	Twins excluded, women recruited from 26 nationwide sites, 10.8% smoking, 11.7% alcohol use, 0.6% IDU	Risk factor analysis	Early ultrasound or LNMP	Average
Tiam *et al.*[38]	Lesotho	Middle	Prospective	6/2014 to 2/2016	1594	Women recruited from 14 mixed setting study centres across 3 districts, 91.6% delivered in a health facility	None	LNMP	Poor
Zash *et al.*[39]	Botswana	Middle	Retrospective	15/8/2014 to 15/8/2016	46267	Twins excluded, women recruited from 8 government hospitals, 6.3% alcohol use or smoking	Regression analysis	LNMP confirmed by ultrasound where possible	Average
Zash *et al.*[40]	Botswana	Middle	Retrospective	15/8/2014 to 15/8/2016	57005	Twins excluded, women recruited from 8 government hospitals, hospital deliveries, 8.3% alcohol or smoking in pregnancy	Regression analysis	LNMP and/or ultrasound (unspecified), or symphysis-fundal height	Average

IDU, illicit drug use; LNMP, last normal menstrual period.

a Details on the inclusion of twins, recruitment centre, urban/rural setting, deliveries at home/hospital, smoking, alcohol use, and IDU were sought and reported here if provided by each study.

The class of ART, specific antiretroviral drugs, timing of ART initiation, and perinatal outcomes analyzed in each study are summarized in Table 2. Seventeen studies (77%) reported data on WHIV receiving NNRTI-based ART (11 studies EFV-based ART [22,26,28–31,34,35,37,40,41], five studies NVP-based ART [25,31–33,40], one study mixed NNRTI-based ART [38], and two studies unspecified [35,37]), 11 studies (50%) reported on protease inhibitor based ART (three studies lopinavir/ritonavir (LPV/r)-based ART [20,25,40], two studies mixed protease inhibitor based ART [21,24], and six studies unspecified [23,27–29,36,38]) and two studies (9%) reported on INSTI-based ART (one study DTG-based ART [41], one study unspecified [36]). Eleven studies reported on WHIV receiving ART initiated preconception, 12 studies reported on antenatal ART initiation, and nine studies reported mixed initiation (Table 2). In total, 19 studies reported PTB, seven VPTB, one sPTB, 10 LBW, four VLBW, two Term LBW, one Preterm LBW, 15 SGA, six VSGA, one stillbirth, and five NND.

**Table 2:** Antiretroviral therapy characteristics and perinatal outcomes.

Study	ART class	Antiretroviral drugs	Timing of ART initiation^a^	Perinatal Outcomes
Azria *et al.*[19]	PI	100% LPV/r-based ART	Mixed initiation	PTB, VPTB, SGA, VSGA, NND
Balogun *et al.*[20]	PI	58.2% LPV/r-based ART 36.4% ATV/r- based ART 5.5% DRV/r- based ART	Mixed initiation	sPTB, SGA
Bengtson *et al.*[21]	NNRTI	100% TDF-FTC/3TC-EFV	Antenatal initiation	PTB, SGA, VSGA
Boer *et al.*[22]	PI	Unspecified	Mixed initiation	PTB
Carceller *et al.*[23]	PI	77.4% NFV-based ART 10.8% INV-based ART 4.4% LPV/r-based ART 7.7% SQV-based ART	Mixed initiation	PTB, Term LBW
Chen *et al.*[24]	92.4% NNRTI	100% ZDV-3TC-NVP	Antenatal and preconception initiation	PTB
	7.6% PI	100% ZDV-3TC-LPV/r		
Dadabhai *et al.*[25]	NNRTI	100% TDF-3TC-EFV	Antenatal and preconception initiation (PTB only), Mixed initiation (other outcomes)	PTB, LBW, Term LBW, Preterm LBW, SGA, VSGA
Gagnon *et al.*[26]	PI	Unspecified	Mixed initiation	PTB, LBW, SGA
Malaba *et al.*[27]	97% NNRTI	100% TDF-3TC-EFV	Antenatal and preconception initiation	PTB, VPTB, LBW, VLBW, SGA
	3% PI	Unspecified		
Malaba *et al.*[28]	97.2% NNRTI	96.7% TDF-3TC-EFV, 0.5% TDF-3TC-NVP, 2.7% other NNRTI-based ART	Antenatal and preconception initiation	PTB, SGA
	2.8% PI	Unspecified		
Mehta *et al.*[29]	NNRTI	95.2% TDF-FTC-EFV, 1.6% TDF-3TC-EFV, 2.1% TDF-FTC-NVP, 0.2% D4T-3TC-NVP, 0.5% other EFV-based ART (D4T-3TC-EFV or ZDV-3TC-EFV) 0.4% other NVP-based ART (ZDV-3TC-NVP or TDF-3TC-NVP)	Mixed initiation	PTB, LBW, SGA, NND
Moodley *et al.*[30]	NNRTI	22.4% D4T-3TC-NVP, 77.6% TDF-FTC-EFV	Mixed initiation	PTB, LBW, SGA
Olagbuji *et al.*[31]	NNRTI	100% ZDV-3TC-NVP	Mixed initiation	LBW
Ramokolo *et al.*[32]	NNRTI	100% TDF-3TC/FTC-NVP	Antenatal and preconception initiation	PTB, LBW, SGA
Rempis *et al.*[33]	NNRTI	98% TDF-3TC-EFV 2% unspecified	Antenatal and preconception initiation	SGA
Santosa *et al.*[34]	95.9% NNRTI	98.2% TDF-FTC/3TC-EFV, 1.8% NVP-based ART	Antenatal and preconception initiation	PTB, VPTB, LBW, VLBW, SGA, VSGA, Stillbirth, NND
Saums *et al.*[35]	34% NNRTI	Unspecified	Mixed initiation	PTB
	54.7% PI			
	10.9% INSTI			
Sebitloane *et al.*[36]	NNRTI	TDF-FTC-NVP/EFV ratio unspecified	Antenatal and preconception initiation	PTB
Snijdewind *et al.*[37]	31.5% NNRTI	Unspecified	Antenatal and preconception initiation	PTB, VPTB, LBW, VLBW, SGA
	66.7% PI			
Tiam *et al.*[38]	NNRTI	86.0% TDF-3TC-EFV 3.9% TDF-3TC-NVP 4.4% ZDV-3TC-EFV 4.6% ZDV-3TC-NVP 1.1% other ART	Antenatal and preconception initiation	PTB, LBW, VLBW, VPTB
Zash *et al.*[39]	92% NNRTI	53.8% EFV-based ART 46.2% NVP-based ART	Preconception initiation	PTB, VPTB, SGA, VSGA, NND
	8% PI	100% LPV/r-based ART		
Zash *et al.*[40]	72.7% NNRTI	100% TDF-FTC-EFV	Antenatal initiation	PTB, VPTB, SGA, VSGA, NND
	27.3% INSTI	100% TDF-FTC-DTG		

3TC, lamivudine; ART, antiretroviral therapy (triple drug therapy); ATV/r, atazanavir/ritonavir; D4T, stavudine; DRV/r, darunavir/ritonavir; DTG, dolutegravir; EFV, efavirenz; FTC, emtricitabine; INSTI, integrase strand transfer inhibitor; INV, indinavir; LBW, low birthweight; LPV/r, lopinavir/ritonavir; NFV, nelfinavir; NND, neonatal death; NNRTI, nonnucleoside reverse transcriptase inhibitor; NVP, nevirapine; PI, protease inhibitor; PTB, preterm birth; SGA, small for gestational age; sPTB, spontaneous preterm birth; SQV, saquinavir; TDF, tenofovir disoproxil fumarate; VLBW, very low birthweight; VPTB, very preterm birth; VSGA, very small for gestational age; ZDV, zidovudine.

a “Antenatal initiation” refers to ART, which was initiated after the estimated date of conception. “Preconception initiation” refers to ART, which was initiated before the estimated date of conception. “Mixed initiation” refers to ART, which includes both preconception or antenatal initiation where the outcome data are not reported separately according to timing of ART initiation. “Preconception and antenatal initiation” refers to studies that report distinct outcome data for each timing of ART initiation (preconception or antenatal).

Perinatal outcomes among WHIV receiving either NNRTI-based ART (Fig. 2a), protease inhibitor based ART (Fig. 2b), or INSTI-based ART (Fig. 2c) were compared with women without HIV (Appendix 3). If two or more studies reported data for the same exposure comparison and outcome, a random-effects meta-analysis was conducted. Subgroup analysis was carried out for studies reporting on WHIV receiving NNRTI-based, who received either EFV-based ART (Fig. 3a) or NVP-based ART (Fig. 3b). Subgroup analysis for specific protease inhibitor and INSTI-based regimens was not possible due to the lack of drug diversity and specification, and data availability. Furthermore, subgroup analyses were conducted according to timing of ART initiation, country income status, and study quality (Appendix 4).

**Fig. 2:**
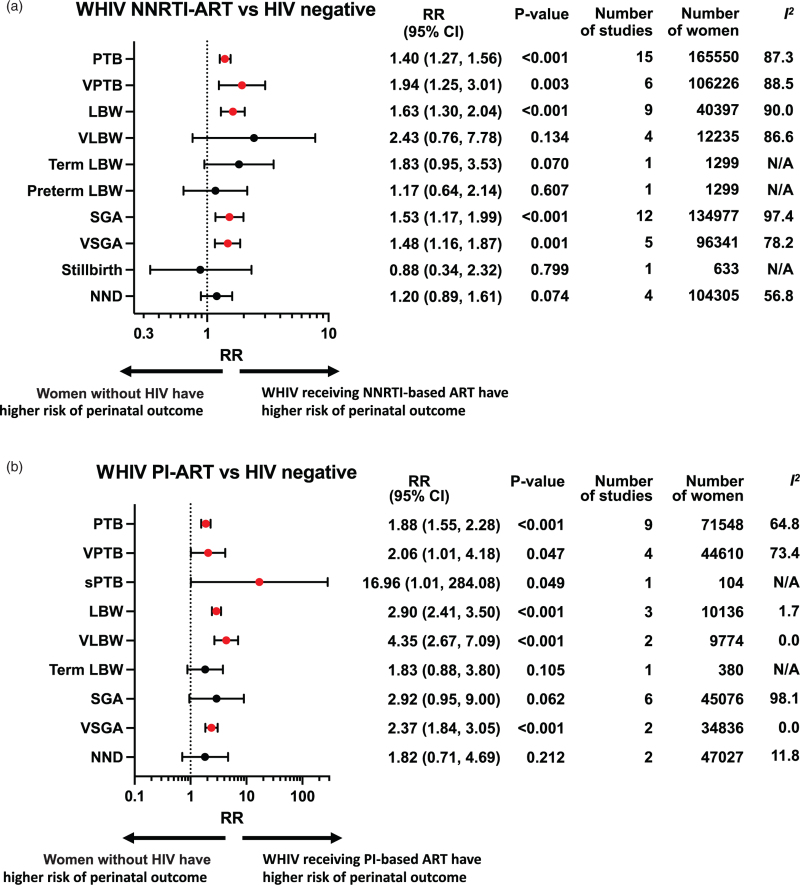
Perinatal outcomes of women with HIV receiving different classes of antiretroviral therapy compared to women without HIV.

Adverse perinatal outcomes associated with WHIV receiving NNRTI-based ART (a), protease inhibitor based ART (b), or INSTI-based ART (c), compared to women without HIV. Results of individual studies or, when two or more studies were available, random-effects meta-analyses are presented. Risk ratios (RRs) and 95% confidence intervals (CIs), p-values, numbers of studies and women included in the analysis of each perinatal outcome, and *I*^*2*^ value for heterogeneity are displayed. Statistically significant (*P* < 0.05) effect estimates are presented with red dots and nonsignificant effects with black dots. Forest plots of the meta-analyses for each perinatal outcome can be found in Appendix 4. ART, antiretroviral therapy (triple drug therapy); INSTI, integrase strand transfer inhibitor; LBW, low birthweight; NND, neonatal death; NNRTI, nonnucleoside reverse transcriptase inhibitor; PI, protease inhibitor; PTB, preterm birth; SGA, small for gestational age; sPTB, spontaneous preterm birth; VLBW, very low birthweight; VPTB, very preterm birth; VSGA, very small for gestational age; WHIV, women with HIV.

**Fig. 2 (Continued.):**
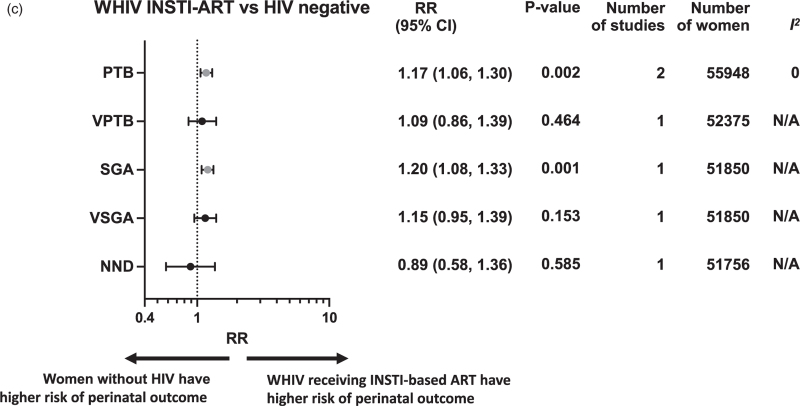
Perinatal outcomes of women with HIV receiving different classes of antiretroviral therapy compared to women without HIV.

Adverse perinatal outcomes associated with WHIV receiving NNRTI-based ART (a), protease inhibitor based ART (b), or INSTI-based ART (c), compared to women without HIV. Results of individual studies or, when two or more studies were available, random-effects meta-analyses are presented. Risk ratios (RRs) and 95% confidence intervals (CIs), p-values, numbers of studies and women included in the analysis of each perinatal outcome, and *I*^*2*^ value for heterogeneity are displayed. Statistically significant (*P* < 0.05) effect estimates are presented with red dots and nonsignificant effects with black dots. Forest plots of the meta-analyses for each perinatal outcome can be found in Appendix 4. ART, antiretroviral therapy (triple drug therapy); INSTI, integrase strand transfer inhibitor; LBW, low birthweight; NND, neonatal death; NNRTI, nonnucleoside reverse transcriptase inhibitor; PI, protease inhibitor; PTB, preterm birth; SGA, small for gestational age; sPTB, spontaneous preterm birth; VLBW, very low birthweight; VPTB, very preterm birth; VSGA, very small for gestational age; WHIV, women with HIV.

**Fig. 3:**
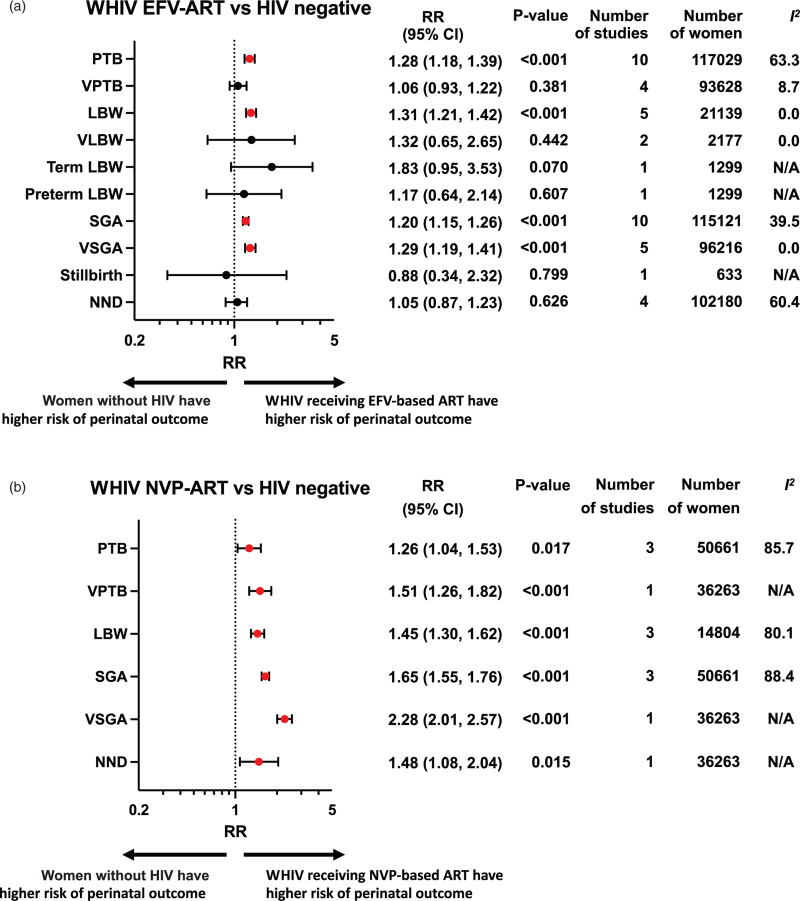
Perinatal outcomes of women with HIV receiving different nonnucleoside reverse transcriptase inhibitor based antiretroviral therapy regimens compared to women without HIV.

Adverse perinatal outcomes associated with WHIV receiving efavirenz (EFV)-based ART (a) or nevirapine (NVP)-based ART (b), compared to women without HIV. Results of individual studies or, when two or more studies were available, random-effects meta-analyses are presented. Risk ratios (RR) and 95% confidence intervals (95% CIs), p-values, numbers of studies and women included in the analysis of each perinatal outcome, and *I*^*2*^ value for heterogeneity are displayed. Statistically significant (*P* < 0.05) effect estimates are presented with red dots and nonsignificant effects with black dots. Forest plots of the meta-analyses for each perinatal outcome are reported in Appendix 4. ART, antiretroviral therapy (triple drug therapy); EFV, efavirenz; LBW, low birthweight; NND, neonatal death; NVP, nevirapine; PTB, preterm birth; SGA, small for gestational age; sPTB, spontaneous preterm birth; VLBW, very low birthweight; VPTB, very preterm birth; VSGA, very small for gestational age; WHIV, women with HIV.

### Women with HIV receiving nonnucleoside reverse transcriptase inhibitor based antiretroviral therapy

A meta-analysis of 15 studies, including 165 550 women, found that WHIV receiving NNRTI-based ART had an increased risk of PTB compared to women without HIV (risk ratio 1.40, 95% CI 1.27–1.56; *P* < 0.001) (Fig. 2a). There was a high level of heterogeneity (*I*^*2*^ = 87.3%), but the Peters’ test found no evidence of small-study effect (*P* = 0.865). Subgroup analysis showed that both EFV-based ART (1.28, 1.18–1.39; *P* < 0.001) (Fig. 3a) and NVP-based ART (1.26, 1.04–1.53; *P* = 0.017) (Fig. 3b) were associated with an increased risk of PTB compared to women without HIV. The increased risk of PTB was seen across ART initiation subgroups, average and poor quality studies, and across LMICs (Appendix 4).

Analysis of six studies, including 106 226 women, found an association between VPTB and NNRTI-based ART (1.94, 1.25–3.01; *P* = 0.003; *I*^*2*^ = 88.5%) (Fig. 2a). In subgroup analysis, NVP-based ART was associated with an increased risk of VPTB (1.51, 1.26–1.82; *P* < 0.001), but not EFV-based ART (1.06, 0.93–1.22; *P* = 0.381) (Fig. 3).

Meta-analysis of nine studies, including 40 397 women, found that WHIV receiving NNRTI-based ART were associated with an increased risk of LBW compared to women without HIV (1.63, 1.30–2.04; *P* < 0.001; *I*^2^ = 90.0.%) (Fig. 2a). This association remained significant for both EFV-based ART (1.31, 1.21–1.42; *P* < 0.001) and NVP-based ART (1.45, 1.30–1.62; *P* < 0.001) (Fig. 3), as well as across subgroups of ART initiation, country income status, and average-quality studies (Appendix 4).

There was no increased risk of VLBW, Term LBW, or Preterm LBW among WHIV receiving NNRTI-based ART compared to women without HIV (Fig. 2a).

Meta-analysis of 12 studies, including 134 977 women, found an increased risk of SGA associated with WHIV receiving NNRTI-based ART, compared to women without HIV (1.53, 1.17–1.99; *P* < 0.001; *I*^*2*^ = 97.4%) (Fig. 2a). There was no evidence of small-study effects (*P* = 0.906). The increased risk of SGA was seen for EFV-based ART (1.20, 1.15–1.26; *P* < 0.001) and NVP-based ART (1.65, 1.55–1.76; *P* < 0.001) (Fig. 3) and across country income status and study quality (Appendix 4).

Similarly, an increased risk of VSGA was found in a meta-analysis of five studies, including 96 341 women, for WHIV receiving NNRTI-based ART (1.48, 1.16–1.87; *P* < 0.001; *I*^*2*^ = 78.2%) (Fig. 2a), including in subgroup analyses of WHIV receiving both EFV-based ART (1.29, 1.19–1.41; *P* < 0.001) and NVP-based ART (2.28, 2.01–2.57; *P* < 0.001) (Fig. 3), and ART initiated preconception and antenatally (Appendix 4).

One study of 633 women compared stillbirth between WHIV on NNRTI-based ART and women without HIV and found no difference in risk (0.88, 0.34–2.32; *P* = 0.799) (Fig. 2a). Four studies, including 104 305 women, found no difference in risk of NND (1.20, 0.89, 1.61; *P* = 0.074) (Fig. 2a).

### Women with HIV receiving protease inhibitor based antiretroviral therapy

Meta-analysis of nine studies, including 71 548 women, found that WHIV receiving protease inhibitor based ART were associated with an increased risk of PTB, compared with women without HIV (1.88, 1.55–2.28; *P* < 0.001; *I*^*2*^ = 64.8%) (Fig. 2b). This association remained significant across all subgroup analyses (Appendix 4).

Analysis of four studies, including 44 610 women, found that WHIV receiving protease inhibitor based ART had an increased risk of VPTB (2.06, 1.01–4.18; *P* = 0.047, *I*^*2*^ = 73.4%) (Fig. 2b), while one study reported an increased risk of sPTB among WHIV receiving protease inhibitor based ART, compared to women without HIV (16.96, 1.01–284.08; *P* = 0.049) (Fig. 2b).

Three studies, including 10 136 women, found an increased risk of LBW among WHIV receiving protease inhibitor based ART, compared to women without HIV (2.90, 2.41–3.50; *P* < 0.001; *I*^*2*^ = 1.7%) (Fig. 2b). This association was significant when protease inhibitor based ART was initiated preconception and antenatally (Appendix 4).

Furthermore, two studies, including 9774 women, found an increased risk of VLBW among WHIV receiving protease inhibitor based ART, compared to women without HIV (4.35, 2.67–7.09; *P* < 0.001; *I*^*2*^ = 0%) (Fig. 2b), irrespective of the timing of ART initiation (Appendix 4).

One study reported Term LBW, which found no difference between WHIV receiving protease inhibitor based ART and women without HIV (1.83, 0.88–3.80; *P* = 0.015) (Fig. 2b).

Six studies, including 45 076 women, found no association between SGA and WHIV receiving protease inhibitor based ART, compared to women without HIV (2.92, 0.95–9.00; *P* = 0.062) (Fig. 2b). There was high heterogeneity (*I*^*2*^ = 98.1%). Interestingly, subgroup analysis found an increased risk of SGA among studies from both LMICs (1.63, 1.34–2.00; *P* < 0.001) and HICs (4.65, 1.08–20.06; *P* = 0.040) (Appendix 4).

Two studies, including 34 836 women, found that WHIV taking protease inhibitor based ART were associated with an increased risk of VSGA, compared to women without HIV (2.37, 1.84–3.05; *P* < 0.001; *I*^*2*^ = 0%) (Fig. 2b).

Two studies, including 47 027 women, found no association of NND with WHIV receiving protease inhibitor based ART, compared to women without HIV (1.82, 0.71–4.69; *P* = 0.212; *I*^*2*^ = 11.8%) (Fig. 2b).

### Women with HIV receiving integrase strand transfer inhibitor based antiretroviral therapy

Meta-analysis of two studies, including 55 948 women, demonstrated an increased risk of PTB among WHIV receiving INSTI-based ART, compared with women without HIV (1.17, 1.06–1.30; *P* = 0.002; *I*^*2*^ = 0%) (Fig. 2c).

Analysis of one study, including 51 850 women, showed an increased risk of SGA among WHIV receiving INSTI-based ART, compared to women without HIV (1.20, 1.08–1.33; *P* = 0.001) (Fig. 2c).

There was no association between WHIV receiving INSTI-based ART and risk of VPTB, VSGA, or NND (Fig. 2c). There were no studies which reported LBW, VLBW, term LBW, Preterm LBW, or stillbirth.

## Discussion

This systematic review and meta-analysis found that WHIV receiving NNRTI-based ART (mainly EFV or NVP) are at increased risk of PTB, VPTB, LBW, SGA, and VSGA, compared to women without HIV. WHIV receiving protease inhibitor based ART (mainly LPV/r or unspecified) are at an increased risk of PTB, VPTB, sPTB, LBW, VLBW, and VSGA, and WHIV receiving INSTI-based ART (mainly DTG) are at an increased risk of PTB and SGA, compared to women without HIV.

Although ART classes were not compared head-to-head, point estimates of relative risk compared to women without HIV were lowest for WHIV receiving INSTI-based ART across all adverse perinatal outcomes with data available. This provides some support toward the WHO recommendation of INSTI-based ART as first line for use in pregnant WHIV, in line with previous work [9,17]. In addition, in the current study, the estimates of relative risk compared to women without HIV were consistently greater across all adverse perinatal outcomes with available data for WHIV receiving protease inhibitor based ART compared to NNRTI-based ART. This suggests that protease inhibitor based therapy has the strongest association with adverse perinatal outcomes. However, findings should be interpreted with caution in view of the different comparator groups of women without HIV and the high levels of heterogeneity among analysed studies.

Our analysis found no significant association between protease inhibitor based ART and risk of SGA, when compared to women without HIV. This contrasts with previous meta-analyses showing protease inhibitor based ART during pregnancy to be associated with increased risks of SGA and VSGA when compared to NNRTI-based ART [16,17,42]. This nonsignificant association between protease inhibitor based ART and risk of SGA is in part attributable to a very high heterogeneity (98.1%) between the six average quality studies, illustrated by the fact that the association was significant in the subgroup analysis of protease inhibitor based ART in both LMICs and HICs. In addition, the point estimate is greater for SGA among WHIV receiving protease inhibitor based ART (risk ratio 2.92, 0.95–9.00), than for NNRTI-based (risk ratio 1.53, 1.17–1.99) or INSTI-based ART (risk ratio 1.20, 1.08–1.33), although the overlapping 95% CIs mean that uncertainty remains regarding comparative differences between ART classes.

This study has several strengths. It is the first systematic review and meta-analysis to compare a comprehensive range of 11 perinatal outcomes between WHIV receiving different classes of ART and women without HIV. Furthermore, this study is large, including 191 857 women across 22 studies and 10 different countries, providing strong evidence for the significant associations found. Of these studies, 15 (68%), including 175 839 (92%) women, were conducted in LMICs, where the majority of WHIV live, increasing the external validity of our conclusions. Outcomes and exposures were predefined to minimize misclassification bias, while a random-effects meta-analysis model was used to account for heterogeneity between studies. Furthermore, by analyzing studies comparing WHIV receiving ART with contemporaneous women without HIV, we reduce the risk of chronological bias that can occur when comparing classes directly, because of novel drug development and confounding improvements in health outcomes over time. Quality assessments, subgroup and sensitivity analyses, as well as assessment of adjustment for confounders and small study effects strengthens the validity of observed associations.

We acknowledge several limitations of this meta-analysis. First, all included studies were observational, precluding our ability to establish causality and making them vulnerable to bias. Apart from HIV status, there were likely medical, psychological, and socioeconomic differences between WHIV and women without HIV, which are not consistently accounted for across included studies. Five studies (23%) did not report a method to assess gestational age, while only four studies used the most accurate method: first trimester ultrasound. Thus, outcomes such as PTB and SGA may have been subject to misclassification bias. There is the possibility of indication bias, related to the fact that protease inhibitor based ART are second-line regimens in many countries, and hence, recipients are more likely to have failed other regimens. Data availability also limits this meta-analysis. No studies reported on triple NRTI-based ART. Only two studies report on INSTI-based ART, including 55 948 women and five perinatal outcomes (PTB, VPTB, SGA, VSGA, NND), with the majority of these outcomes only reported by one study. This limits the scope and accuracy of this part of our analysis and necessitates further study in the future, especially since INSTI-based therapy is currently recommended as first line [9]. Similarly, there are insufficient studies reporting perinatal outcomes for specific INSTI-based ART regimens (such as raltegravir, cabotegravir, bictegravir, and elvitegravir) and protease inhibitor based ART regimens (such as LPV/r, atazanavir/ritonavir, and darunavir/ritonavir), precluding further subgroup analysis of individual drugs. Where multiple studies reported on the same exposure comparison and perinatal outcome, there was generally a high level of heterogeneity and relatively large CIs in the analyses. This is likely due to differences in study populations and settings.

The mechanisms through which both HIV and ART are associated with adverse perinatal outcomes such as PTB, LBW, and SGA are poorly understood. It is thought that systemic immune activation persists despite successful inhibition of viral replication by ART [43]. Several studies in pregnant women have observed a decline in various innate immune cell frequencies in early HIV infection that do not recover with ART and appear to be associated with perinatal outcomes, such as PTB [44–46]. Relatedly, previous work suggests altered cytokine profiles between WHIV receiving ART and women without HIV throughout pregnancy, which may also be related to perinatal outcomes [47]. Other studies have shown that an antiangiogenic placental state is associated with adverse perinatal outcomes, including sPTB, SGA, and stillbirth, in WHIV on ART [48,49]. Some studies have shown that antenatal exposure to protease inhibitor based ART was associated with uteroplacental and decidual dysfunction, decreased progesterone levels and alterations in oestradiol and prolactin during pregnancy, which correlate with adverse birth outcomes [50–53]. Meanwhile, an RCT reported that NNRTI-based ART was associated with lower oestradiol levels, SGA and LBW [54].

Overall, there is a clear need for more large well conducted prospective observational studies of perinatal outcomes among pregnant WHIV receiving different ART drugs and regimens. This is particularly important for individual INSTI-based ART drugs and regimens, including dolutegravir, raltegravir, bictegravir, and elvitegravir, as well as long-acting injectable cabotegravir, as INSTI-based ART is currently recommended as first-line, as well as for novel therapies such as the first-in-class HIV-1 capsid inhibitor lenacapavir, and mAbs [55,56]. It is essential that studies collect and report detailed information about ART regimens, timing of ART initiation, and outcomes, and correct for potential confounders. It is also crucial to have long-term follow-up studies to assess the effects of intrauterine ART exposure on the growth and neurodevelopment of HIV-exposed uninfected children [57].

While it is clear that ART in pregnancy has important benefits for maternal health and reduces vertical and horizontal HIV transmission, pregnant WHIV receiving any class of ART remain at increased risk of a wide range of adverse perinatal outcomes compared to women without HIV. More research is urgently needed to optimize ART in pregnancy and improve perinatal outcomes for WHIV.

## Acknowledgements

M.H. and L.T. selected relevant studies, conducted the meta-analyses, subgroup, and sensitivity analyses, interpreted the data, and wrote the first draft of the manuscript. L.T. and M.H. contributed equally to this study. M.H., L.T., C.P., H.S., M.K., and Z.B. screened the literature search results for relevant manuscripts and assessed their eligibility, verified and extracted data, and conducted methodological quality assessments. S.K. designed and conducted the literature search. J.H. conceived, designed and coordinated the study, developed the systematic review protocol, assisted with the literature search, assessment of eligibility of manuscripts, data extraction, methodological quality assessment, designed the meta-analysis plan, interpreted the data, and wrote the manuscript. All authors had full access to all the data in the study and had final responsibility for the decision to submit for publication.

### Conflicts of interest

There are no conflicts of interest.

## Supplementary Material

Supplemental Digital Content
