# Supplementary material for: Adverse perinatal outcomes associated with different classes of antiretroviral drugs in pregnant women with HIV
Source: AIDS. 2024 Oct 15;39(2):162–74. doi: 10.1097/QAD.0000000000004032 (PMC11676599; doi:10.1097/QAD.0000000000004032)

Appendix

Adverse perinatal outcomes associated with different classes of antiretroviral drugs in pregnant women living with HIV: a systematic review and meta-analysis.

TABLE OF CONTENTS

Appendix 1: Literature search strategies 5

Appendix 2: Quality assessment of studies 9

Appendix 2.1: Adapted Newcastle-Ottawa quality assessment tool 9

Appendix 2.2: Classification of studies according to quality assessment........ 10

Appendix 2.3: Quality assessment of studies included in the systematic review and meta-analysis 11

Appendix 2.4: Confounding factors adjusted for in included studies........ 13

Appendix 3: Random-effects meta-analyses of risk of perinatal outcomes associated with pregnancies in women living with HIV receiving different ART regimens compared to HIV negative women........ 15

Appendix 3.1: Women living with HIV receiving NNRTI-based ART compared to HIV-negative women 15

Figure 3.1.1 Preterm birth in women living with HIV receiving NNRTI-based ART compared to HIV-negative women 15

Figure 3.1.2 Very Preterm birth in women living with HIV receiving NNRTI-based ART compared to HIV-negative women 15

Figure 3.1.3 Low birthweight in women living with HIV receiving NNRTI-based ART compared to HIV-negative women 16

Figure 3.1.4 Very low birthweight in women living with HIV receiving NNRTI-based ART compared to HIV-negative women 16

Figure 3.1.5 Small for gestational age in women living with HIV receiving NNRTI-based ART compared to HIV-negative women........16

Figure 3.1.6 Very Small for gestational age in women living with HIV receiving NNRTI-based ART compared to HIV-negative women........17

Figure 3.1.7 Neonatal death in women living with HIV receiving NNRTI-based ART compared to HIV-negative women 17

Table 3.1.8 Term low birthweight, Preterm Low Birthweight and Stillbirth in women living with HIV receiving NNRTI-based ART compared to HIV-negative women........17

Appendix 3.2: Women living with HIV receiving PI-based ART compared to HIV-negative women.. 18

Figure 3.2.1 Preterm birth in women living with HIV receiving PI-based ART compared to HIV-negative women 18

Figure 3.2.2 Very Preterm birth in women living with HIV receiving PI-based ART compared to HIV-negative women 18

Figure 3.2.3 Low birthweight in women living with HIV receiving PI-based ART compared to HIV-negative women 19

Figure 3.2.4 Very low birthweight in women living with HIV receiving PI-based ART compared to HIV-negative women 19

Figure 3.2.5 Small for gestational age in women living with HIV receiving PI-based ART compared to HIV-negative women 19

Figure 3.2.6 Very small for gestational age in women living with HIV receiving PI-based ART compared to HIV-negative women...20

Figure 3.2.7 Neonatal death in women living with HIV receiving PI-based ART compared to HIV-negative women 20

Table 3.2.8 Spontaneous Preterm birth and Term low birthweight in women living with HIV receiving PI-based ART compared to HIV-negative women...20

Appendix 3.3: Women living with HIV receiving INSTI-based ART compared to HIV-negative women 21

Figure 3.3.1 Preterm birth in women living with HIV receiving INSTI-based ART compared to HIV-negative women 21

Table 3.3.2 Very preterm birth, small for gestational age, very small for gestational age, and neonatal death in women living with HIV receiving INSTI-based ART compared to HIV-negative women 21

Appendix 4: Subgroup analyses 22

Appendix 4.1: Timing of ART initiation 22

4.1.1: Subgroup analysis based on Preconception and Antenatal Initiation: Risk Ratios..........................22

4.1.2: Subgroup analysis based on Preconception and Antenatal Initiation: Number of studies and women analysed 23

4.1.3: Subgroup analysis based on Preconception and Antenatal Initiation: I^2 values:..............................24

Appendix 4.2: Country Income Status 25

4.2.1: Subgroup analysis based on High Income Countries and Low- and Middle-Income Countries: Risk Ratios:..25

4.2.2: Subgroup analysis based on High Income Countries and Low- and Middle-Income Countries: Number of studies and women analysed..26

4.2.3: Subgroup analysis based on High Income Countries and Low- and Middle-Income Countries: I^2 values: 27

Appendix 4.3: Study Quality: good, average and low quality studies 29

4.3.1: Subgroup analysis based on study quality: Risk ratios 29

4.3.2: Subgroup analysis based on study quality: Number of studies and women analysed 30

4.3.3: Subgroup analysis based on study quality: I^2 values 32

Appendix 4.4: Drug regimen analysis 33

4.4.1: Subgroup analysis of studies involving Efavirenz or Nevirapine containing NNRTI-based ART: Risk ratios, number of women analysed, and I^2 values 33

Appendix 5: Sensitivity analyses 35

Appendix 6: Funnel plots 36

Figure 6.1: Preterm birth in women living with HIV receiving NNRTI-based ART vs HIV negative women 36

Figure 6.2: Very preterm birth in women living with HIV receiving NNRTI-based ART vs HIV negative women 36

Figure 6.3: Low birthweight in women living with HIV receiving NNRTI-based ART vs HIV negative women 37

Figure 6.4: Very low birthweight in women living with HIV receiving NNRTI-based ART vs HIV negative women 37

Figure 6.5: Small for gestational age in women living with HIV receiving NNRTI-based ART vs HIV negative women 38

Figure 6.6: Very small for gestational age in women living with HIV receiving NNRTI-based ART vs HIV negative women 38

Figure 6.7: Neonatal death in women living with HIV receiving NNRTI-based ART vs HIV negative women 39

Figure 6.8: Preterm birth in women living with HIV receiving PI-based ART vs HIV negative women 40

Figure 6.9: Very Preterm birth in women living with HIV receiving PI-based ART vs HIV negative women 40

Figure 6.10 Low birthweight in women living with HIV receiving PI-based ART vs HIV negative women 41

Figure 6.11: Very low birthweight in women living with HIV receiving PI-based ART vs HIV negative women 41

Figure 6.12: Small for gestational age in women living with HIV receiving PI-based ART vs HIV negative women 42

Figure 6.13: Very small for gestational age in women living with HIV receiving PI-based ART vs HIV 42

Figure 6.14: Neonatal death in women living with HIV receiving PI-based ART vs HIV negative 43

Figure 6.15: PTB in women living with HIV receiving INSTI-based ART vs HIV negative 44

APPENDIX 1: LITERATURE SEARCH STRATEGIES

Search strategy for “pregnancy outcomes” AND “HIV” OR “Antiretroviral therapy/ARVs”.

Database and platform: MEDLINE (Ovid MEDLINE® Epub Ahead of Print, In-Process & Other Non-Indexed Citations, Ovid MEDLINE Daily and Ovid MEDLINE®) 1946 to present (via OVID)

Last search date: 14 July 2023

1. Pregnancy Outcome/ or exp Pregnancy Complications, Infectious/

2. ((pregnancy or gestational or fetal or foetal or obstetric$) adj1 (outcome$ or complication$ or consequence$ or characteristic$ or event$ or result$ or problem$ or morbidit$ or sequelae)).ti,ab.

3. ((labor or labour or birth or delivery or neonate or newborn or "new-born" or "new born") adj1 (outcome$ or complication$ or consequence$ or characteristic$ or event$ or result$ or problem$ or morbidit$ or sequelae)).ti,ab.

4. ((infant or reproductive or prelabour or prelabor or "pre-labour" or "pre-labor" or intrauterine or "intra-uterine") adj1 (outcome$ or complication$ or consequence$ or characteristic$ or event$ or result$ or problem$ or morbidit$ or sequelae)).ti,ab.

5. ((antenatal or "ante-natal" or prenatal or "pre-natal" or perinatal or "peri-natal" or neonatal or "neo-natal" or postnatal or "post-natal") adj1 (outcome$ or complication$ or consequence$ or characteristic$ or event$ or result$ or problem$ or morbidit$ or sequelae)).ti,ab.

6. ((antepartum or "ante-partum" or intrapartum or "intra-partum" or peripartum or "peri-partum" or postpartum or "post-partum") adj1 (outcome$ or complication$ or consequence$ or characteristic$ or event$ or result$ or problem$ or morbidit$ or sequelae)).ti,ab.

7. Premature Birth/ or exp Fetal Membranes, Premature Rupture/ or Obstetric Labor, Premature/ or Infant, Extremely Premature/ or Infant, Premature/

8. (prematurity or "gestational age at birth" or "gestational age at delivery" or PTB or PTBs or VPTB or VPTBs or "pre-terms" or preterms or PTL or PTLs or VPTL or VPTLs or PTD or PTDs or VPTD or VPTDs or PROM or PPROM).ti,ab.

9. (("pre-term" or preterm or premature) adj2 (labour$ or labor$ or infant or deliver$ or birth$)).ti,ab.

10. ((preterm or "pre-term" or premature) adj1 rupture adj3 membrane$).ti,ab.

11. Fetal Growth Retardation/ or Infant, Low Birth Weight/ or Infant, Very Low Birth Weight/ or Infant, Extremely Low Birth Weight/ or Infant, Small for Gestational Age/

12. ((intrauterine or "intra-uterine" or fetal or foetal) adj1 growth adj1 (restrict$ or retardation)).ti,ab.

13. (SGA or SFGA or IUGR or FGR or "small for gestational age" or "small-for-gestational-age" or "small-for-gestational age" or "small for gestation" or "small-for-gestation").ti,ab.

14. (VSGA or "very-small-for-gestational-age" or "very-small-for-gestational age" or SFD or "small for dates" or "small-for-dates" or "weight for dates" or "weight for gestational age" or "weight for age at delivery" or "weight at delivery").ti,ab.

15. ("birthweight for dates" or "birthweight for gestational age" or "birthweight for age at delivery" or "birth weight for dates" or "birth weight for gestational age" or "birth weight for age at delivery" or "birth-weight for dates" or "birth-weight for gestational age" or "birth-weight for age at delivery").ti,ab.

16. (LBW or "low BW" or "low birth weight" or "low birth-weight" or "low-birth weight" or "low-birth-weight" or "low birthweight" or "low-birthweight" or "lower BW" or "lower birth weight" or "lower birth-weight" or "lower-birth weight" or "lower-birth-weight" or "lower birthweight" or "lower-birthweight").ti,ab.

17. ("reduced birth weight" or "reduced birthweight" or "reduced birth-weight" or VLBW or "very-low birthweight" or "very-low birth weight" or "very-low birth-weight" or "very-low-birthweight" or "very-low-birth-weight" or ELBW or "extremely-low birthweight" or "extremely-low

birth weight" or "extremely-low birth-weight" or "extremely-low-birthweight" or "extremely-low-birth-weight").ti,ab.

18. Stillbirth/ or Fetal Death/

19. (stillbirth$ or "still birth$" or stillborn$ or "still born$" or abortion$ or miscarriage$).ti,ab.

20. ((pregnancy or gestational or fetal or foetal or obstetric$ or labor or labour or birth) adj1 (death$ or loss$ or demise$ mortalit$)).ti,ab.
21. ((delivery or neonate or newborn or "new-born" or "new born" or infant or reproductive or prelabour or prelabor or "pre-labour" or "pre-labor") adj1 (death$ or loss$ or demise$ mortalit$)).ti,ab.

22. ((intrauterine or "intra-uterine" or antenatal or ante-natal or prenatal or "pre-natal" or perinatal or "peri-natal" or neonatal or "neo-natal" or postnatal or "post-natal" or antepartum or "ante-partum" or intrapartum or "intra-partum" or peripartum or "peri-partum" or postpartum or "post-partum") adj1 (death$ or loss$ or demise$ mortalit$)).ti,ab.

23. 1 or 2 or 3 or 4 or 5 or 6 or 7 or 8 or 9 or 10 or 11 or 12 or 13 or 14 or 15 or 16 or 17 or 18 or 19 or 20 or 21 or 22

24. HIV/ or HIV Seropositivity/ or HIV Infections/ or HIV-2/ or HIV-1/ or AIDS Serodiagnosis/ or Acquired Immunodeficiency Syndrome/ or AIDS Arteritis, Central Nervous System/ or AIDS-Associated Nephropathy/ or AIDS Dementia Complex/ or AIDS-Related Opportunistic Infections/ or Lymphoma, AIDS-Related/

25. (HIV or "HIV/AIDS" or PLHIV or PLWHA or WLHIV or WLWHA or "HIV-1" or "HIV-type-1" or "HTLV III" or "HTLV-III" or "HTLV type III" or "HTLV-type-III" or LAV or "HTLV-III-LAV" or "LAV-HTLV-III" or "HIV-2" or "HIV-type-2" or "HIV-II" or "HTLV-IV" or "LAV-2").ti,ab.

26. ("HIV-positive" or "HIV-1-positive" or "HIV-2-positive" or "HIV-infected" or "HIV-1-infected" or "HIV-type-1-infected" or "HTLV III-infected" or "HTLV-III-infected" or "HTLV type III-infected" or "HTLV-type-III-infected" or "LAV-infected").ti,ab.

27. ("HTLV-III-LAV-infected" or "LAV-HTLV-III-infected" or "HIV-2-infected" or "HIV-type-2-infected" or "HIV-II-infected" or "HTLV-IV-infected" or "LAV-2-infected" or "HIV-infection$" or "HIV-1-infection$" or "HIV-type-1-infection$" or "HTLV III-infection$" or "HTLV-III-infection$" or "HTLV type III-infection$" or "HTLV-type-III-infection$").ti,ab.

28. ("LAV-infection$" or "HTLV-III-LAV-infection$" or "LAV-HTLV-III-infection$" or "HIV-2-infection$" or "HIV-type-2-infection$" or "HIV-II-infection$" or "HTLV-IV-infection$" or "LAV-2-infection$" or "Human Immunodeficiency Virus$" or "Human Immune Deficiency Virus$").ti,ab.

29. ("Human T Cell Lymphotropic Virus Type III" or "Human T-Cell Lymphotropic Virus Type III" or "Human T Lymphotropic Virus Type III" or "Human T-Lymphotropic Virus Type III" or "Human T Lymphotropic Virus Type IV" or "Human T-Lymphotropic Virus Type IV" or "Human T Cell Leukemia Virus Type III" or "Human T-Cell Leukemia Virus Type III").ti,ab.

30. ("Lymphadenopathy-Associated Virus$" or "Lymphadenopathy Associated Virus$" or AIDS or "Acquired Immune Deficiency Syndrome" or "Acquired Immunodeficiency Syndrome").ti,ab.

31. 24 or 25 or 26 or 27 or 28 or 29 or 30

32. exp Anti-HIV Agents/ or exp HIV Fusion Inhibitors/ or exp HIV Integrase Inhibitors/ or exp HIV Protease Inhibitors/ or HIV Reverse Transcriptase/ or Reverse Transcriptase Inhibitors/

33. ((antiretroviral or "anti-retroviral" or antiviral$ or "anti-viral$" or "anti-HIV" or "anti-HIV-1") adj1 (treatment$ or therap$ or regimen$ or drug$ or agent$)).ti,ab.

34. (("anti-HIV-2" or "anti-AIDS") adj1 (treatment$ or therap$ or regimen$ or drug$ or agent$)).ti,ab.

35. (HAART or "HAART-exposed" or "HAART-treated" or "Mega-HAART" or ARV or ARVs or cARV or cARVs or "ARV-exposed" or "ARV-treated" or "combination-ARV" or "combination-ARVs" or "combined-ARV" or "combined-ARVs").ti,ab.

36. (ART or "Multi-ART" or "Triple-ART" or ART or "ART-exposed" or "ART-treated" or "combination-ART" or "combined-ART" or "sc-ART" or "short-course-antiretroviral therap$" or "short-course-anti-retroviral therap$").ti,ab.

37. (combin$ adj (treatment$ or therap$ or regimen$ or drug$ agent$)).ti,ab.

38. (monotherap$ or "mono-therap$" or "dual therap$" or "dual drug therap$" or bitherap$).ti,ab.

39. (PI or PIs or "PI-based" or "boosted-PI" or "PI-containing" or "PI-therap$" or "PI-treatment$" or

"PI-regimen$" or "Ritonavir-boosted" or "protease inhibitor$" or NRTI or NRTIs or "NRTI-based" or "NRTI-containing" or "NRTI-therap$" or "NRTI-treatment$" or "NRTI-regimen$" or "nucleoside reverse transcriptase inhibitor$" or "nucleoside analog reverse transcriptase inhibitor$").ti,ab.

40. (NNRTI or NNRTIs or "NNRTI-based" or "NNRTI-containing" or "NNRTI-therap$" or "NNRTI-treatment$" or "NNRTI-regimen$" or "non nucleoside reverse transcriptase inhibitor$" or "non-nucleoside reverse transcriptase inhibitor$" or "nonnucleoside reverse transcriptase inhibitor$" or "non nucleoside analog reverse transcriptase inhibitor$" or "non-nucleoside analog reverse transcriptase inhibitor$" or "nonnucleoside analog reverse transcriptase inhibitor$").ti,ab.

41. (NtRTI or NtRTIs or "NtRTI-based" or "NtRTI-containing" or "NtRTI-therap$" or "NtRTI-treatment$" or "NtRTI-regimen$" or "nucleotide reverse transcriptase inhibitor$" or "nucleotide analog reverse transcriptase inhibitor$" or "fusion inhibitor$" or "CCR5 receptor antagonist$" or "integrase inhibitor$" or "maturation inhibitor$" or "entry inhibitor$").ti,ab.

42. Didanosine/ or Delavirdine/ or Emtricitabine/ or Lamivudine/ or Nevirapine/ or Rilpivirine/ or Stavudine/ or Tenofovir/ or Zidovudine/ or Atazanavir Sulfate/ or Darunavir/ or Ritonavir/ or Lopinavir/ or Nelfinavir/ or Saquinavir/ or Enfuvirtide/ or Maraviroc/ or Raltegravir Potassium/ or Indinavir/ or Zalcitabine/ or Cobicistat/

43. "Efavirenz, Emtricitabine, Tenofovir Disoproxil Fumarate Drug Combination"/ or "Emtricitabine, Rilpivirine, Tenofovir Drug Combination"/ or "Emtricitabine, Tenofovir Disoproxil Fumarate Drug Combination"/ or "Elvitegravir, Cobicistat, Emtricitabine, Tenofovir Disoproxil Fumarate Drug Combination"/

44. (Abacavir or ABC or Didanosine or ddI or Emtricitabine or FTC or Lamivudine or 3TC or Stavudine or d4T or Tenofovir or TFV or TDF or TAF or Zidovudine or AZT or ZDV or Delavirdine or DLV or Efavirenz or EFV or Etravirine or ETR or Nevirapine or NVP or Rilpivirine or RPV or Atazanavir or ATV or "Atazanavir/Ritonavir" or "ATV/r" or Darunavir or DRV or "Darunavir/Ritonavir" or "DRV/r" or Fosamprenavir or FPV or "Fosamprenavir/Ritonavir" or "FPV/r" or temsavir or TMR).ti,ab.

45. (Indinavir or IDV or "Indinavir/Ritonavir" or "IDV/r" or Lopinavir or LPV or "Lopinavir/Ritonavir" or "LPV/r" or Nelfinavir or NFV or "Nelfinavir/Ritonavir" or "NFV/r" or Ritonavir or RTV or Saquinavir or SQV or "Saquinavir/Ritonavir" or "SQV/r" or Tipranavir or TPV or "Tipranavir/Ritonavir" or "TPV/r" or Enfuvirtide or "T-20" or Maraviroc or MVC or Raltegravir or RAL or Elvitegravir or EVG or Zalcitabine or ddC or Combivir or Trizivir or Kaletra or Epzicom or Kivexa or Truvada or Atripla).ti,ab.

46. ("Integrase strand transfer inhibitor" or Dolutegravir or DTG or Tivicay or Isentress or Vitekta or "Formyl peptide receptor 1" or Fuzeon or FPR1 or ENF or Seizentry or Celsentri or Ziagen or Videx or Emtriva or Coviracil or Zerit or Viread or Vemlidy or Retrovir or Azidothymidine or "Diarylpyrimidine analogue" or Rescriptor or Sustiva or Intelence or TMC125 or DAPY or Viramune or Edurant or TMC278).ti,ab.

47. (Reyataz or APV or Agenerase or Prezista or Lexiva or Telzir or Crixivan or ABT-378 or Norvir or Viracept or AG1343 or Invirase or Fortovase or Aptivus or "Rilpivirine plus dolutegravir" or "RPV/DTG" or "Raltegravir plus lamivudine" or "RAL/3TC" or "Abacavir plus lamivudine plus dolutegravir" or "ABC/3TC/DTG" or "Emtricitabine plus tenofovir alafenamide" or "FTC/TAF" or "Emtricitabine plus rilpivirine plus tenofovir alafenamide" or "FTC/RPV/TAF" or "Atazanavir plus cobicistat" or "ATV/COBI" or "Darunavir plus cobicistat" or "DRV/COBI" or Juluca or Dutrebis or Stribild or Triumeq or Odefsey or Complera or Descovy or Genvoya or Evotaz or Prezcobix).ti,ab.

48. (Cobicistat or "cobicistat-boosted" or COBI or Rezolsta or QUAD or Epivir or Temixys or Cimduo or Selzentry or Doravirine or DOR or Pifeltro or "Ibalizumab-uiyk" or Hu5A8 or IBA or Ibalizumab or "TMB-355" or "TNX-355" or Trogarzo or Bictegravir or BIC or "Bictegravir, Emtricitabine, Tenofovir Alafenamide" or "bictegravir sodium/emtricitabine/tenofovir alafenamide fumarate" or "BIC/FTC/TAF" or Biktarvy or "tenofovir alafenamide fumarate" or "darunavir ethanolate, cobicistat, emtricitabine, tenofovir alafenamide fumarate" or "DRV/COBI/FTC/TAF").ti,ab.

49. (Symtuza or "Dolutegravir and lamivudine" or "dolutegravir sodium/lamivudine" or "DTG/3TC" or Dovato or "Doravirine, lamivudine, and tenofovir disoproxil fumarate" or "doravirine/lamivudine/tenofovir disoproxil fumarate" or "DOR/3TC/TDF" or Delstrigo or "Efavirenz, lamivudine, and tenofovir disoproxil fumarate" or "EFV/3TC/TDF" or Symfi or "Symfi Lo" or "Elvitegravir, cobicistat, emtricitabine, and tenofovir alafenamide fumarate" or

"elvitegravir/cobicistat/emtricitabine/tenofovir alafenamide fumarate" or "EVG/COBI/FTC/TAF" or Genvoya or "TMC-114" or "TMC114" or Dideoxyinosine or Racivir or Heptovir or Hepitec).ti,ab.

50. (Zerut or Estavudina or Sanilvudine or Apropovir or Stocrin or Zrivada or Aluvia or Aluviran or Pentafuside or Symtuza or "Efavirenz, Lamivudine, Tenofovir Disoproxil Fumarate drug combination" or "Lopinavir-Ritonavir drug combination").ti,ab.

51. (NRTTI or NRTTIs or "NRTTI-based" or "NRTTI-containing" or "NRTTI-therap$" or "NRTTI-treatment$" or "NRTTI-regimen$" or "nucleoside reverse transcriptase translocation inhibitor" or islatravir or ISL or "MK-8591" or "portmanteau inhibitor$" or "capsid inhibitor$" or "CAI" or lenacapavir or "LEN" or "GS-CA1" or leronlimab or albuvirtide or Eviplera or "rilpivirine, emtricitabine and tenofovir disoproxil fumarate" or "attachment inhibitor" or "gp120 attachment inhibitor" or Rukobia or fostemsavir or "FTV" or INSTI or INSTIs or Vocabria or cabotegravir or "CAB" or cabenuva or Tybost or "INSTI-based" or "INSTI-containing" or "INSTI-therap$" or "INSTI-treatment$" or "INSTI-regimen$").ti,ab.

52. 32 or 33 or 34 or 35 or 36 or 37 or 38 or 39 or 40 or 41 or 42 or 43 or 44 or 45 or 46 or 47 or 48 or 49 or 50 or 51

53. 31 or 52
54. 23 and 53
55. limit 54 to yr="2020-2023"

APPENDIX 2: QUALITY ASSESSMENT OF STUDIES

APPENDIX 2.1: ADAPTED NEWCASTLE-OTTAWA QUALITY ASSESSMENT TOOL

A study can be awarded a maximum of one point (for items indicated with an asterisk) for each numbered criterion within the "Selection" and "Outcome" categories.

Selection (maximum 4 points)

1) Representativeness of the exposed cohort.

   a) Truly representative of the pregnant population in the community. *

   b) Somewhat representative of the pregnant population in the community.

   c) Selected group of users, e.g. nurses, volunteers, teenage mothers.

   d) No description of the derivation of the cohort.

2) Selection of the comparator cohort.

   a) The comparator cohort is drawn from the same community as the exposed cohort. *

   b) The comparator cohort is drawn from a different source than the exposed cohort.

   c) No description of the derivation of the comparator cohort.

3) Ascertainment of exposure.

   a) ART intake monitored as part of study. *

   b) ART intake confirmed from secure medical records (e.g. hospital records). *

   c) Structured interview-participant reported ART intake.

   d) Written self-report.

   e) No description.

4) Demonstration that outcome of interest was not present at start of study.

   a) Yes. *

   b) No.

Comparability (maximum 2 points)

1) Comparability of cohorts on the basis of the analysis. In the analysis:

   a) Study controls for BMI, smoking, parity, and maternal age. *

   b) Study controls for one or more additional factors: e.g. prior history of adverse pregnancy outcome, maternal hypertension, anaemia, illicit drug or alcohol use in pregnancy. *

   c) Confounding factors not controlled for.

Outcome (maximum 3 points)

1) Ascertainment of outcome.

a) Outcome was confirmed following clinical observation of outcome by clinician, midwife or trained birth attendant. *

b) Medical records. *

c) Self-report.

d) No description.

2) Method used to assess gestational age.

a) Gestational age was determined according to early ultrasound (<14 weeks). *

b) Gestational age was determined by: late ultrasound (≥14 weeks' gestation) or last normal menstrual period or neonatal assessment, e.g. Ballard score, or a combination of these methods.

c) No description.

3) Follow up of cohorts

a) Complete follow up - all subjects accounted for. *

b) Subjects lost to follow up unlikely to introduce bias, i.e. < 20 % lost to follow up. *

c) Follow up rate < 80% (lost to follow-up > 20%).

d) No description.

APPENDIX 2.2: CLASSIFICATION OF STUDIES ACCORDING TO QUALITY ASSESSMENT

Good Quality	9 points – all requirements met
Average Quality	3 points in "Selection" and 3 points in "Outcome" sections
	≥2 points in the "Selection" and "Outcome" sections, as well as ≥1 point in the "Comparability" section.
Poor Quality	< 2 points in the "Selection" and/or "Outcome" sections.
	2 points in the "Selection" and "Outcome" sections, but no points in the "Comparability" section.

APPENDIX 2.3: QUALITY ASSESSMENT OF STUDIES INCLUDED IN THE SYSTEMATIC REVIEW AND META-ANALYSIS

Study	Representativeness of the exposed cohort	Selection of comparator cohort	Ascertainment of exposure	Demonstration that outcome of interest was not present at the start of the study	Comparability of cohorts on the basis of the analysis	Ascertainment of outcome	Method to assess gestational age	Follow up of cohorts	Total quality assessment
**Azria et al (2009)**	Truly representative*	Same community as the exposed cohort*	ART monitored as part of study*	No	Study controls for one or more additional confounding factors*	Medical records*	First day of LNMP, corrected if needed by routine first trimester ultrasound*	Complete follow up*	Average
**Balogun et al (2018)**	Somewhat representative	Same community as the exposed cohort*	ART intake monitored as part of study*	Yes*	Study controls for one or more additional confounding factors*	Clinical observation*	LNMP confirmed by ultrasound (unspecified)	Complete follow up*	Average
**Bengtson et al (2020)**	Truly representative*	Same community as the exposed cohort*	ART intake monitored as part of study*	Yes*	Confounding factors not controlled for	Clinical observation*	Ultrasound (unspecified), LNMP or symphysis-fundal height	Complete follow up*	Poor
**Boer et al (2006)**	Truly representative*	Different source than the exposed cohort	No description	No	Study controls for one or more additional confounding factors*	No description	LNMP confirmed by first trimester ultrasound*	Complete follow up*	Poor
**Carceller et al (2009)**	Truly representative*	Same community as exposed cohort*	Medical records*	No	Confounding factors not controlled for	Medical records*	No description	<20% lost to follow up*	Poor
**Chen et al (2012)**	Truly representative*	Same community as exposed cohort*	Medical records*	No	Study controls for one or more additional confounding factors*	Medical records*	LNMP, symphysis-fundal height or ultrasound (unspecified)	<20% lost to follow up*	Average
**Dadabhai et al (2019)**	Somewhat representative	Same community as exposed cohort*	Medical records*	Yes*	Study controls for one or more additional confounding factors*	Clinical observation*	Ballard score and LNMP	Complete follow up*	Average
**Gagnon et al (2016)**	Truly representative*	Same community as exposed cohort*	Medical records*	No	Study controls for one or more additional confounding factors*	Medical records*	First trimester ultrasound or conception date by assisted reproduction if these were available*	Complete follow up*	Average
**Malaba et al (2017)**	Truly representative*	Same community as exposed cohort*	Self-reported	Yes*	Study controls for one or more additional confounding factors*	Medical records*	LNMP and symphysis-fundal height	<20% lost to follow up*	Average
**Malaba et al (2018)**	Truly representative*	Same community as exposed cohort*	ART intake monitored as part of study*	Yes*	Study controls for one or more additional confounding factors*	Clinical observation*	LNMP and symphysis-fundal height	Complete follow up*	Average
**Mehta et al (2019)**	Truly representative*	Same community as exposed cohort*	Medical records*	No	Study controls for one or more additional confounding factors*	Clinical observation*	LNMP with ultrasound (unspecified)	Complete follow up*	Average
**Moodley et al (2016)**	Truly representative*	Same community as exposed cohort*	Medical records*	No	Study controls for one or more additional confounding factors*	Medical records*	LNMP and/or ultrasound (unspecified)	<20% lost to follow up*	Average

**Olagbuji et al (2010)**	Somewhat representative	Same community as exposed cohort*	No description	Yes*	Study controls for one or more additional confounding factors*	No description	No description	No description	Poor
**Ramokolo et al (2017)**	Truly representative*	Same community as exposed cohort*	Self-reported	No	Study controls for one or more additional confounding factors*	Medical records*	LNMP	Complete follow up*	Average
**Rempis et al (2017)**	Somewhat representative	Same community as exposed cohort*	Self-reported	No	Study controls for one or more additional confounding factors*	Medical records*	No description	<20% lost to follow up.*	Poor
**Santosa et al (2019)**	Truly representative*	Same community as the exposed cohort*	Medical records*	Yes*	Study controls for BMI, smoking, parity, and maternal age *, and one or more additional confounding factors*	Clinical observation*	Ultrasound <14 weeks*	Complete follow up*	Good
**Saums et al (2019)**	Truly representative*	Same community as exposed cohort*	Medical records*	No	Study controls for one or more additional confounding factors*	Medical records*	No description	Complete follow up*	Average
**Sebitloane et al (2017)**	Truly representative *	Same community as exposed cohort*	Medical records*	No	Confounding factors not controlled for	Medical records*	No description	Complete follow up*	Poor
**Snijdewind et al (2018)**	Truly representative*	Same community as exposed cohort*	Medical records*	No	Study controls for one or more additional confounding factors*	Medical records*	Early ultrasound or LNMP	Complete follow up*	Average
**Tiam et al (2019)**	Truly representative*	Same community as the exposed cohort*	Medical records*	Yes*	Confounding factors not controlled for	Structured interviews with nurses and medical records*	LNMP	<20% lost to follow up*	Poor
**Zash et al (2017)**	Truly representative*	Same community as exposed cohort*	Medical records*	No	Study controls for one or more additional confounding factors*	Medical records*	LNMP confirmed by ultrasound where possible	Complete follow up*	Average
**Zash et al (2018)**	Truly representative*	Same community as exposed cohort*	Medical records*	No	Study controls for one or more additional confounding factors*	Medical records*	LNMP and/or ultrasound (unspecified), or symphysis-fundal height	Complete follow up*	Average

Abbreviations: ART= antiretroviral therapy, LNMP= last normal menstrual period.

APPENDIX 2.4: CONFOUNDING FACTORS ADJUSTED FOR IN INCLUDED STUDIES

Study	Methods to assess confounding factors	Regression analysis: confounders corrected for	Risk factor analysis: demographics not significant	Matching
**Azria et al (2009)**	Risk factor analysis, matching	-	History of preterm delivery, tobacco smoking during pregnancy, history of illicit drug injection	For each HIV+ infected woman included in the study, two non-referred controls with confirmed HIV- tests, singleton pregnancy, delivered during the same period in the same department and matched by maternal age (+/- 1 year), parity (nulliparous or multiparous) and geographical origin.
**Balogun et al (2018)**	Risk factor analysis, matching	-	Maternal age, pre-pregnancy BMI, race, parity	Matching between HIV+ and HIV– women was performed on the basis of race, maternal age (±5 years), parity (0, 1, or >1), and body mass index (BMI) (<25 or >25 kg/m2)
**Bengtson et al (2020)**	None	-	No P-value reported for demographics	-
**Boer et al (2006)**	Regression analysis, matching	Maternal age, mode of delivery, parity, ART use in first trimester, maternal CD4 count nadir only	-	Matching HIV- to HIV+: date of expected delivery within 0.5 year, maternal age (±3 years, in most cases ±1 year), parity (nulliparous or multiparous), ethnicity (black, including creole, white or other), singleton or twin
**Carceller et al (2009)**	None	-	-	-
**Chen et al (2012)**	Regression analysis, risk factor analysis	CD4 count in pregnancy, advanced maternal age, nulliparity, maternal hypertension in pregnancy and anaemia	Nationality, education, parity, antenatal care received, syphilis, alcohol, smoking, CD4 count,	-
**Dadabhai et al (2019)**	Regression analysis	Maternal age, gravidity, previous pregnancy losses and adverse outcomes, maternal education (years of schooling), BMI at enrolment, anaemia, electricity	-	-
**Gagnon et al (2016)**	Regression, risk factor analysis	Conditional logistic regression model: Ethnicity, previous preterm birth and medical history of severe illness	Age, smoking, alcohol, illicit drug use, STD	-
**Malaba et al (2017)**	Regression, risk factor analysis	Maternal age, maternal height, parity, previous PTD, CD4 count and viral load	Socio-economic status, height, CD4 count	-

**Malaba et al (2018)**	Regression analysis	Age, parity, BMI, previous PTB	-	-
**Mehta et al (2019)**	Risk factor analysis	-	Education, previous adverse pregnancy outcome, pre-existing diabetes, epilepsy status.	-
**Moodley et al (2016)**	Regression, risk factor analysis	Year age group, mode of delivery, HIV status	Maternal age, year, mode of delivery, CD4 count	-
**Olagbuji et al (2010)**	Risk factor analysis	-	Maternal age (mean and age distribution), marital status	-
**Ramokolo et al (2017)**	Risk factor analysis	-	Syphilis serology, tuberculosis, maternal age, parity, household food insecurity, infant gender, maternal education	-
**Rempis et al (2017)**	Risk factor analysis	-	Maternal age, no of persons in household, income, socio-economic status, travel distance to hospital, referral, grand multiparity, hypertension, MIP reported, anaemia, ANC attendance, no of ANC visits	-
**Santosa et al (2019)**	Regression, risk factor analysis	Maternal age, smoking, alcohol consumption, pregnancy BMI, parity and history of adverse perinatal outcomes	Married/cohabiting, occupation, smoked during pregnancy, alcohol during pregnancy, pregnancy BMI, history of prior adverse perinatal outcomes, gestational age at enrolment	-
**Saums et al (2019)**	Risk factor analysis	-	Parity, history of diabetes, hypertension, diabetes, obesity, IDU	-
**Sebitloane et al (2017)**	None	-	-	-
**Snijdewind et al (2018)**	Risk factor analysis	-	Regions of origin, smoking, alcohol, IDU, mode of delivery	-
**Tiam et al (2019)**	None	-	-	-
**Zash et al (2017)**	Regression analysis	Maternal age, gravidity, low educational attainment	-	-
**Zash et al (2018)**	Regression analysis	Maternal age, gravidity, education	-	-

Abbreviations: ANC=antenatal clinic, ART= antiretroviral therapy, BMI= body mass index, ART= antiretroviral therapy, HIV= human immunodeficiency virus, IDU= illicit drug use, MIP=malaria in pregnancy, PTB=preterm birth, PTD=preterm delivery, STD= sexually transmitted disease.

APPENDIX 3: RANDOM-EFFECTS META-ANALYSES OF RISK OF PERINATAL OUTCOMES ASSOCIATED WITH PREGNANCIES IN WOMEN LIVING WITH HIV RECEIVING DIFFERENT ART REGIMENS COMPARED TO HIV NEGATIVE WOMEN.

Forest plots showing random-effects meta-analyses of risk of perinatal outcomes associated with pregnancies in women living with HIV receiving NNRTI-based ART, PI-based ART, and INSTI-based ART compared to HIV-negative women. Risk ratio (RR) and 95% confidence intervals (CIs).

APPENDIX 3.1: WOMEN LIVING WITH HIV RECEIVING NNRTI-BASED ART COMPARED TO HIV-NEGATIVE WOMEN

FIGURE 3.1.1 PRETERM BIRTH IN WOMEN LIVING WITH HIV RECEIVING NNRTI-BASED ART COMPARED TO HIV-NEGATIVE WOMEN

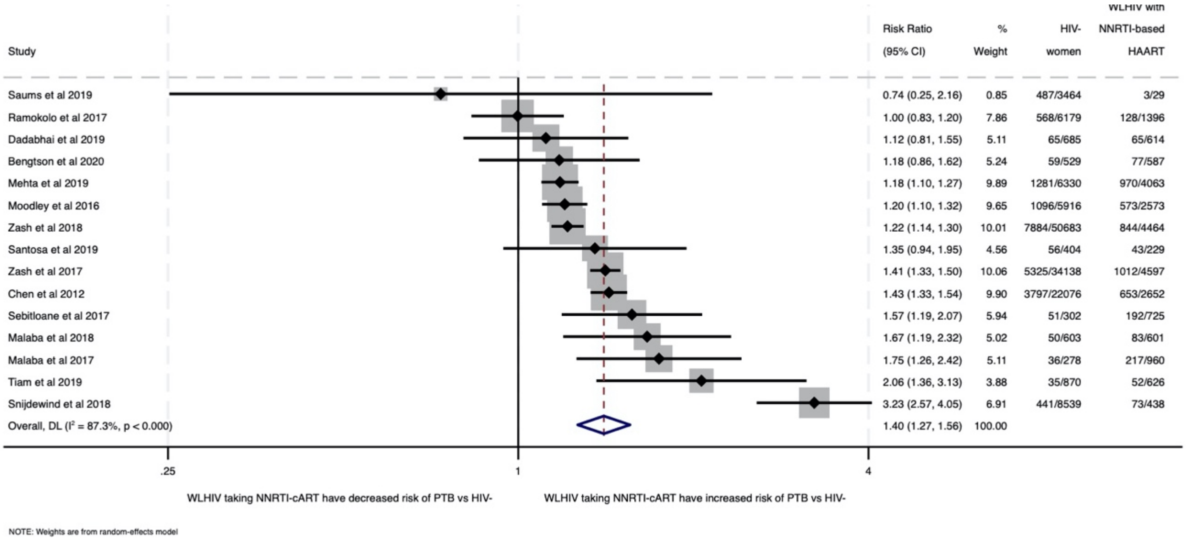

Test of H0: no small-study effects P = 0.865

FIGURE 3.1.2 VERY PRETERM BIRTH IN WOMEN LIVING WITH HIV RECEIVING NNRTI-BASED ART COMPARED TO HIV-NEGATIVE WOMEN

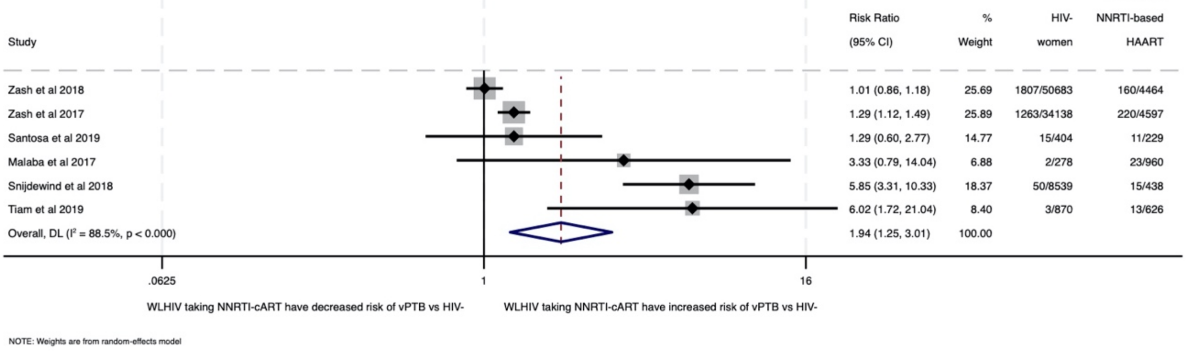

FIGURE 3.1.3 LOW BIRTHWEIGHT IN WOMEN LIVING WITH HIV RECEIVING NNRTI-BASED ART COMPARED TO HIV-NEGATIVE WOMEN

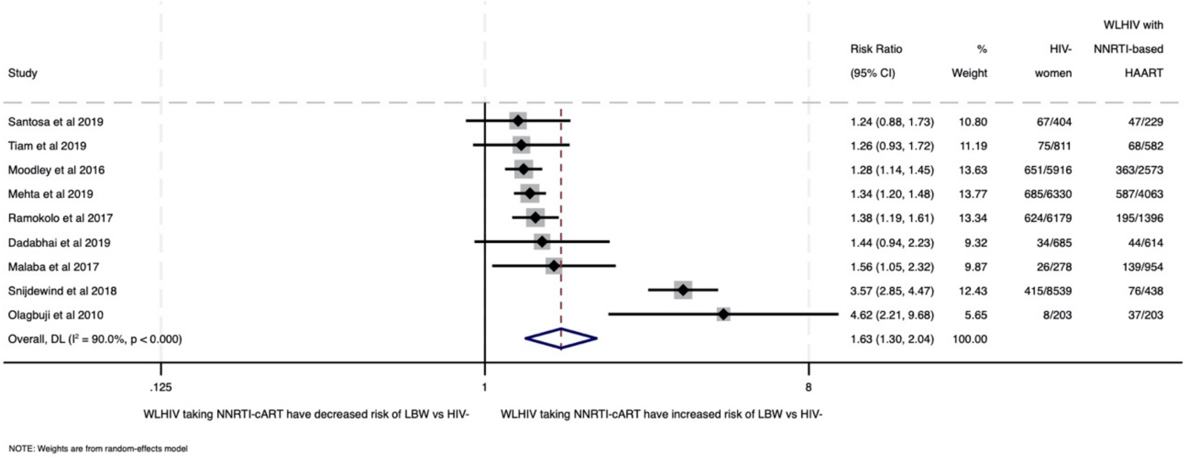

FIGURE 3.1.4 VERY LOW BIRTHWEIGHT IN WOMEN LIVING WITH HIV RECEIVING NNRTI-BASED ART COMPARED TO HIV-NEGATIVE WOMEN

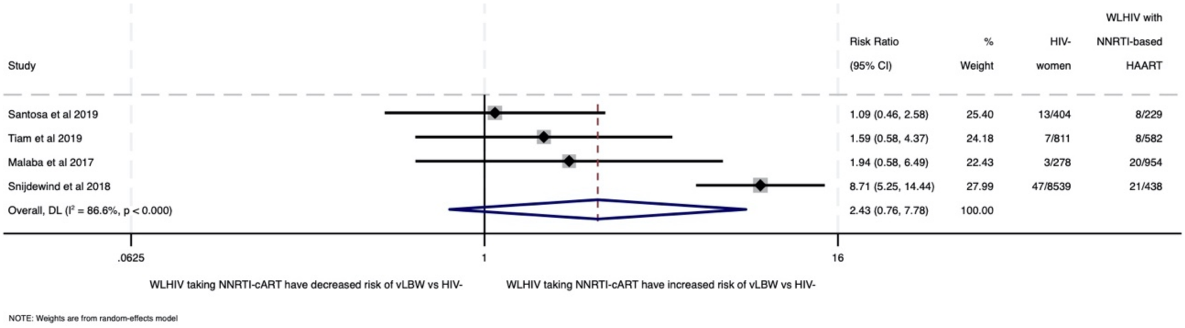

FIGURE 3.1.5 SMALL FOR GESTATIONAL AGE IN WOMEN LIVING WITH HIV RECEIVING NNRTI-BASED ART COMPARED TO HIV-NEGATIVE WOMEN

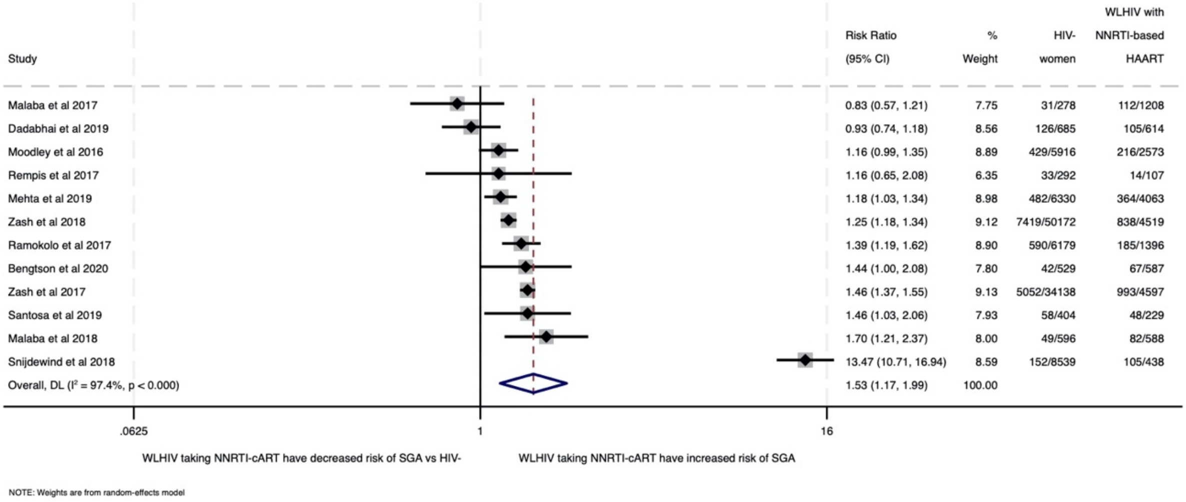

Test of H0: no small-study effects P = 0.906

Figure 3.1.6 Very Small for gestational age in women living with HIV receiving NNRTI-based ART compared to HIV-negative women

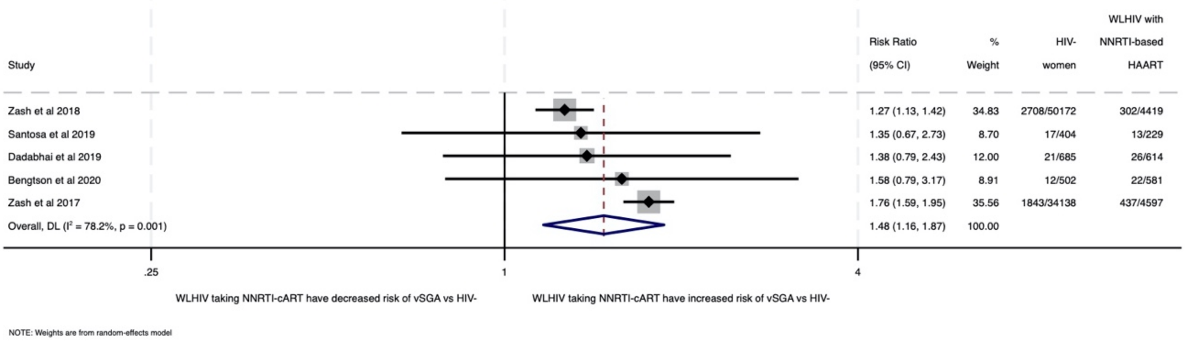

Figure 3.1.7 Neonatal death in women living with HIV receiving NNRTI-based ART compared to HIV-negative women

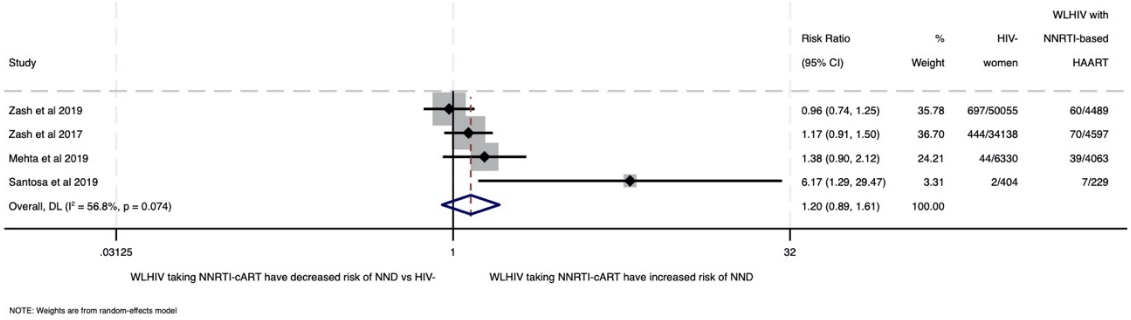

TABLE 3.1.8 Term low birthweight, Preterm Low Birthweight and Stillbirth in women living with HIV receiving NNRTI-based ART compared to HIV-negative women

Outcome	Study	RR	95% CI	P-value	WLHIV receiving NNRTI-based ART	HIV-negative
Term LBW	Dadabhai 2019	1.83	0.95, 3.53	P=0.070	23/614	14/685
Preterm LBW	Dadabhai 2019	1.17	0.64, 2.14	P=0.607	21/614	20/685
Stillbirth	Santosa 2019	0.88	0.34, 2.32	P=0.799	6/229	12/404

Appendix 3.2: Women living with HIV receiving PI-based ART compared to HIV-negative women

Figure 3.2.1 Preterm birth in women living with HIV receiving PI-based ART compared to HIV-negative women

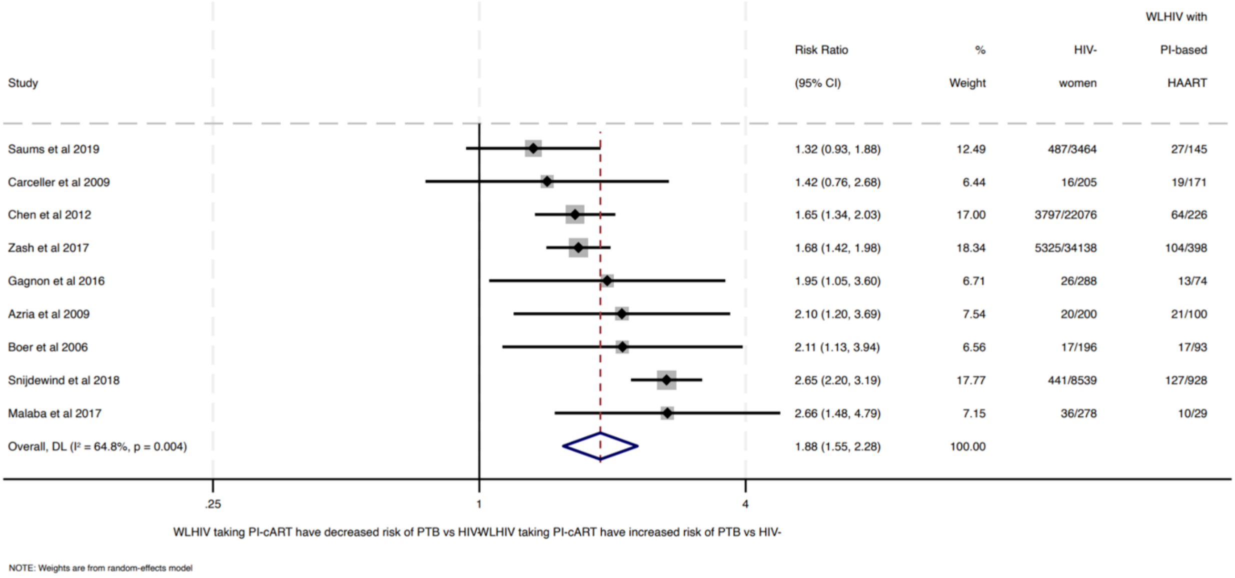

Figure 3.2.2 Very Preterm birth in women living with HIV receiving PI-based ART compared to HIV-negative women

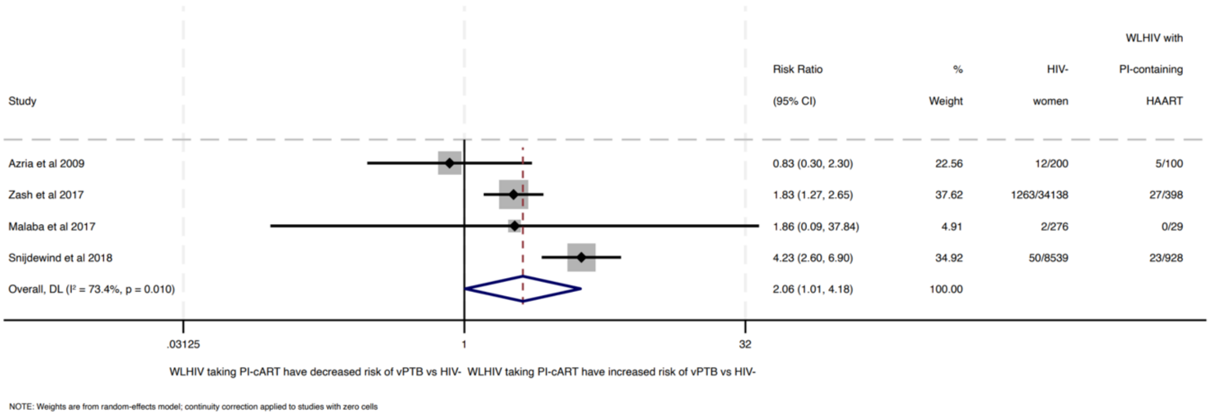

FIGURE 3.2.3 LOW BIRTHWEIGHT IN WOMEN LIVING WITH HIV RECEIVING PI-BASED ART COMPARED TO HIV-NEGATIVE WOMEN

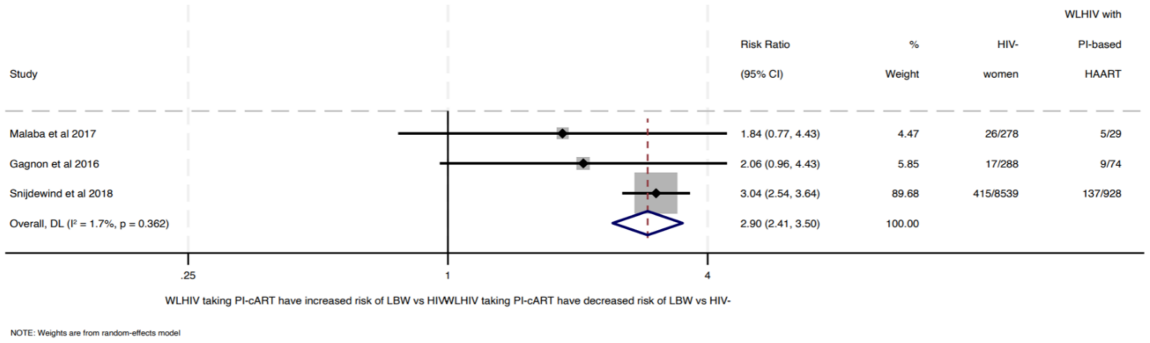

FIGURE 3.2.4 VERY LOW BIRTHWEIGHT IN WOMEN LIVING WITH HIV RECEIVING PI-BASED ART COMPARED TO HIV-NEGATIVE WOMEN

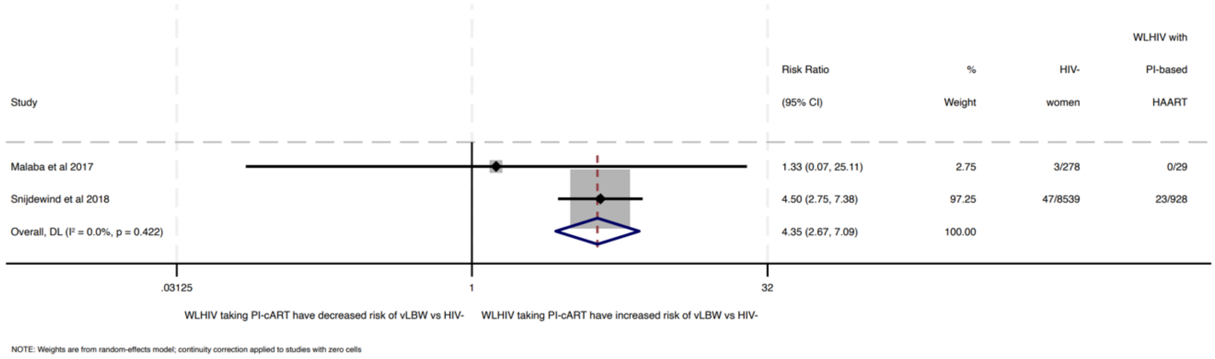

FIGURE 3.2.5 SMALL FOR GESTATIONAL AGE IN WOMEN LIVING WITH HIV RECEIVING PI-BASED ART COMPARED TO HIV-NEGATIVE WOMEN

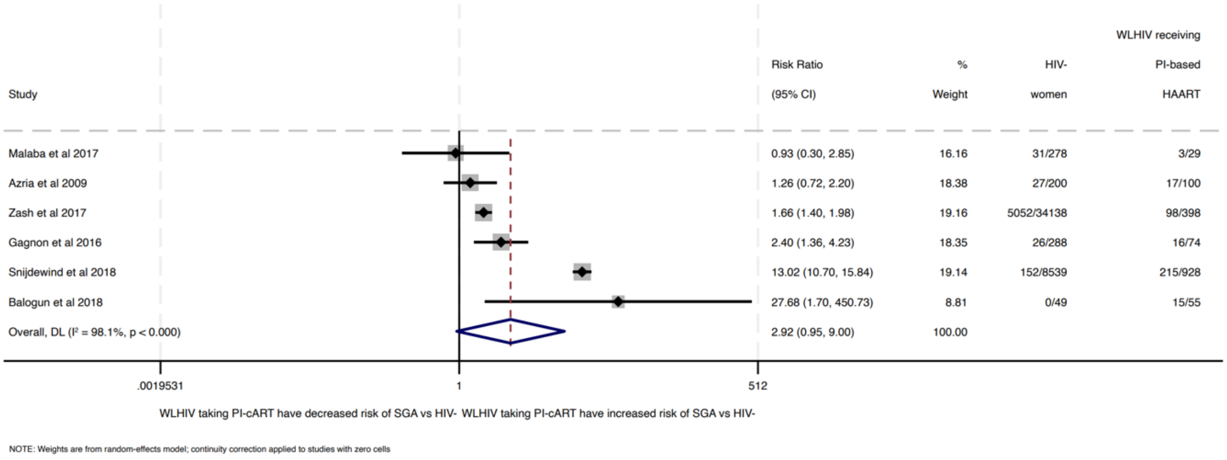

FIGURE 3.2.6 VERY SMALL FOR GESTATIONAL AGE IN WOMEN LIVING WITH HIV RECEIVING PI-BASED ART COMPARED TO HIV-NEGATIVE WOMEN

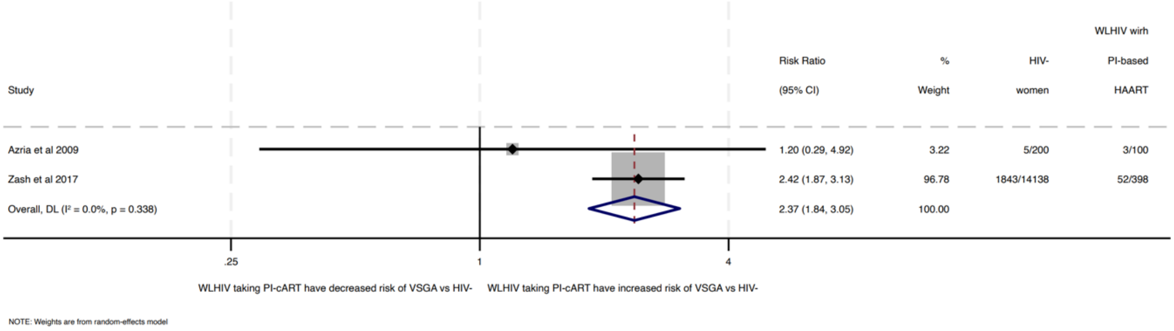

FIGURE 3.2.7 NEONATAL DEATH IN WOMEN LIVING WITH HIV RECEIVING PI-BASED ART COMPARED TO HIV-NEGATIVE WOMEN

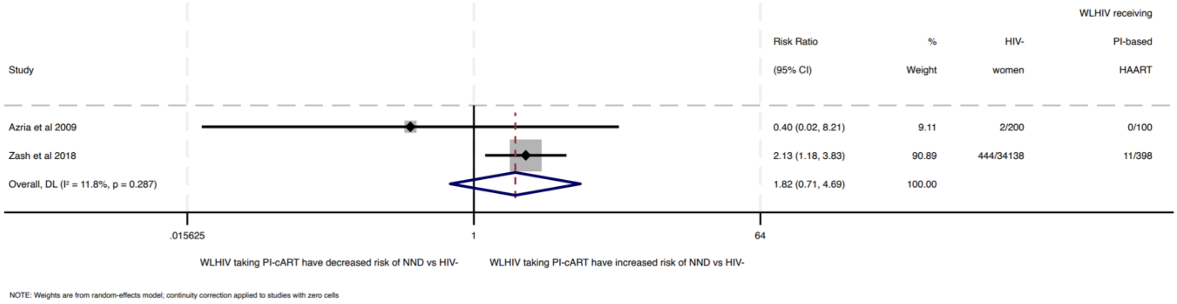

TABLE 3.2.8 SPONTANEOUS PRETERM BIRTH AND TERM LOW BIRTHWEIGHT IN WOMEN LIVING WITH HIV RECEIVING PI-BASED ART COMPARED TO HIV-NEGATIVE WOMEN

Outcome	Study	RR	95% CI	P-value	WLHIV receiving NNRTI-based ART	HIV-negative
Spontaneous Preterm birth (sPTB)	Balogun 2018	16.96	1.01, 284.06	P=0.049	9/55	0/49
Term LBW	Carceller 2009	1.83	0.88, 3.80	P=0.105	17/174	11/206

APPENDIX 3.3: WOMEN LIVING WITH HIV RECEIVING INSTI-BASED ART COMPARED TO HIV-NEGATIVE WOMEN

FIGURE 3.3.1 PRETERM BIRTH IN WOMEN LIVING WITH HIV RECEIVING INSTI-BASED ART COMPARED TO HIV-NEGATIVE WOMEN

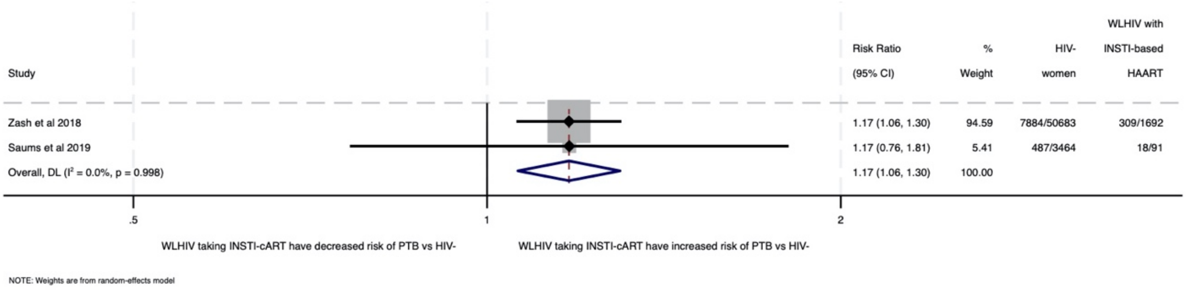

TABLE 3.3.2 VERY PRETERM BIRTH, SMALL FOR GESTATIONAL AGE, VERY SMALL FOR GESTATIONAL AGE, AND NEONATAL DEATH IN WOMEN LIVING WITH HIV RECEIVING INSTI-BASED ART COMPARED TO HIV-NEGATIVE WOMEN

Outcome	Study	RR	95% CI	P-value	WLHIV receiving NNRTI-based ART	HIV-negative
Very Preterm birth (vPTB)	Zash 2018	1.09	0.86, 1.39	P=0.464	66/1692	1807/50683
SGA	Zash 2018	1.20	1.08, 1.33	P=0.001	297/1678	7419/50172
vSGA	Zash 2018	1.15	0.95, 1.39	P=0.153	104/1678	2708/50172
NND	Zash 2018	0.89	0.58, 1.36	P=0.585	21/1701	697/50055

APPENDIX 4: SUBGROUP ANALYSES

APPENDIX 4.1: TIMING OF ART INITIATION

4.1.1: SUBGROUP ANALYSIS BASED ON PRECONCEPTION AND ANTENATAL INITIATION: RISK RATIOS

	Timing of Initiation	Perinatal outcomes							
		**PTB**	**VPTB**	**LBW**	**VLBW**	**SGA**	**VSGA**	**Stillbirth**	**NND**
		RR (95%CI) p-value	RR (95%CI) p-value	RR (95%CI) p-value	RR (95%CI) p-value	RR (95%CI) p-value	RR (95%CI) p-value	RR (95%CI) p-value	RR (95%CI) p-value
**WLHIV NNRTI-ART vs HIV-negative women**	Preconception	1.78 (1.40, 2.27) 0.000	2.80 (0.91, 8.65 0.074	1.84 (1.11, 3.05 0.019	3.65 (1.23, 10.88) 0.020	1.60 (0.84, 3.06) 0.154	1.76 (1.60, 1.95) 0.000	0.42 (0.025, 6.883) 0.540	1.54 (0.49, 4.85) 0.458
	Antenatal	1.43 (1.18, 1.74) 0.000	1.70 (0.72, 4.02) 0.231	1.68 (1.24, 2.28) 0.001	2.07 (0.89, 4.80) 0.091	1.80 (1.07, 3.04) 0.026	1.28 (1.14, 1.43) 0.000	0.89 (0.12, 6.63) 0.906	2.62 (0.26, 26.78) 0.417
**WLHIV PI-ART vs HIV-negative women**	Preconception	2.40 (1.66, 3.46) 0.000	3.11 (1.07, 9.08) 0.038	3.02 (1.73, 5.26) 0.000	4.35 (2.04, 9.29) 0.000	3.08 (0.50, 18.97) 0.225	2.42 (1.87, 3.13) 0.000		2.13 (1.18. 3.83) 0.012
	Antenatal	1.87 (1.18, 2.98 0.008	6.33 (0.95, 42.17) 0.056	2.81 (2.27, 3.48) 0.000	5.34 (2.00, 14.24) 0.001	8.04 (2.03, 31.74) 0.003			
**WLHIV INSTI-ART vs HIV-negative women**	Antenatal	1.17 (1.06, 1.30 0.002	1.09 (0.86, 1.39) 0.464			1.20 (1.08, 1.33) 0.001	1.15 (0.95, 1.39) 0.153		0.89 (0.58, 1.37) 0.585

A RR > 1 indicates increased risk of a perinatal outcome associated with the specified ART class. For example, preconception NNRTI-ART is associated with an increased risk of preterm birth compared to HIV negative women (RR 1.78 95% CI 1.40, 2.27, p=0.000).

Abbreviations: ART= antiretroviral therapy, HIV= human immunodeficiency virus, PI = protease inhibitor, NNRTI = non-nucleoside reverse transcriptase inhibitor, INSTI = integrase strand transfer inhibitor, LBW= low birthweight, NND= neonatal death, PTB= preterm birth, RR= risk ratio, SGA= small for gestational age, sPTB= spontaneous preterm birth, VLBW= very low birthweight, VPTB= very preterm birth, VSGA= very small for gestational age.

4.1.2: Subgroup analysis based on Preconception and Antenatal Initiation: Number of studies and women analysed

	Timing of Initiation	**Perinatal outcomes**							
		**PTB**	**VPTB**	**LBW**	**VLBW**	**SGA**	**VSGA**	**Stillbirth**	**NND**
		Number of studies (Number of women analysed) Events/total for each group	Number of studies (Number of women analysed) Events/total for each group	Number of studies (Number of women analysed) Events/total for each group	Number of studies (Number of women analysed) Events/total for each group	Number of studies (Number of women analysed) Events/total for each group	Number of studies (Number of women analysed) Events/total for each group	Number of studies (Number of women analysed) Events/total for each group	Number of studies (Number of women analysed) Events/total for each group
**WLHIV NNRTI-ART vs HIV-negative women**	Preconception	9<br>(58811)<br>1370/6813<br>HIV+<br>6627/51998<br>HIV-	4<br>(48421)<br>239/5062<br>HIV+<br>1330/43359<br>HIV-	5<br>(17541)<br>202/1330<br>HIV+<br>1207/1621<br>HIV-	4<br>(10746)<br>28.714<br>HIV+<br>70/9732<br>HIV-	8<br>(62436)<br>1207/6094<br>HIV+<br>6394/56342<br>HIV-	2<br>(39177)<br>440/4625<br>HIV+<br>1860/34542<br>HIV-	1<br>(442)<br>0/38<br>HIV+<br>12/404<br>HIV-	2<br>(39177)<br>71/4635<br>HIV+<br>446/34542<br>HIV-
	Antenatal	10<br>(77365)<br>1397/8293<br>HIV+<br>9245/69072<br>HIV-	4<br>(65379)<br>182/5475<br>HIV+<br>1874/59904<br>HIV-	5<br>(18372)<br>302/2161<br>HIV+<br>1207/16211<br>HIV-	4<br>(11413)<br>24/1381<br>HIV+<br>70/9732<br>HIV-	8<br>(74308)<br>1211/7319<br>HIV+<br>8374/66989<br>HIV-	3<br>(56116)<br>327/5038<br>HIV+<br>2737/51078<br>HIV-	1<br>(442)<br>1/38<br>HIV+<br>12/404<br>HIV-	2<br>(54986)<br>62/4527<br>HIV+<br>699/50459<br>HIV-
**WLHIV PI-ART vs HIV-negative women**	Preconception	4<br>(65774)<br>179/743<br>HIV+<br>9599/65031<br>HIV-	3<br>(42298)<br>37/658<br>HIV+<br>1315/41640<br>HIV-	2<br>(9114)<br>53/297<br>HIV+<br>67/8817<br>HIV-	2<br>(9114)<br>7/297<br>HIV+<br>50/8817<br>HIV-	3<br>(43650)<br>178/695<br>HIV+<br>5235/42955<br>HIV-	1<br>(34536)<br>52/398<br>HIV+<br>1843/34138<br>HIV-		1<br>(34536)<br>11/398<br>HIV+<br>444/34138<br>HIV-
	Antenatal	3<br>(3927)<br>126/838<br>HIV+<br>4274/30893<br>HIV-	2<br>(9477)<br>13/8817<br>HIV+<br>52/660<br>HIV-	2<br>(9477)<br>90/660<br>HIV+<br>441/8817<br>HIV-	2<br>(9477)<br>16/660<br>HIV+<br>50/8817<br>HIV-	2<br>(9477)<br>138/660<br>HIV+<br>183/8817<br>HIV-			

**WLHIV INSTI-ART vs HIV-negative women**	Antenatal	1<br>(6760)<br>309/1692<br>HIV+<br>7884/50683<br>HIV-	1<br>(6760)<br>66/1692<br>HIV+<br>1807/50683<br>HIV-			1<br>(6695)<br>297/1678<br>HIV+<br>7419/50172<br>HIV-	1<br>(6709)<br>104/1692<br>HIV+<br>2708/50172<br>HIV-		1<br>(51756)<br>21/1701<br>HIV+<br>697/50055<br>HIV-

4.1.3: Subgroup analysis based on Preconception and Antenatal Initiation: I^2 values:

	Timing of Initiation	**Perinatal outcomes**							
		**PTB**	**VPTB**	**LBW**	**VLBW**	**SGA**	**VSGA**	**Stillbirth**	**NND**
		I^2<br>(95%CI)<br>(p value)	I^2<br>(95%CI)<br>(p value)	I^2<br>(95%CI)<br>(p value)	I^2<br>(95%CI)<br>(p value)	I^2<br>(95%CI)<br>(p value)	I^2<br>(95%CI)<br>(p value)	I^2<br>(95%CI)<br>(p value)	I^2<br>(95%CI)<br>(p value)
**WLHIV NNRTI-ART vs HIV-negative women**	Preconception	86.0<br>(0.0, 95.1)<br>0.000	89.5<br>97.4)<br>0.000	90.6<br>(0.0, 97.0)<br>0.000	74.8<br>(0.0, 92.3)<br>0.008	97.4<br>(40.9, 99.2)<br>0.000	0.0<br>(0.0, 0.0)<br>0.916	N/A	35.0<br>(0.0, 87.1)<br>0.215
	Antenatal	78.2<br>(0.0-91.8)<br>0.000	66.3<br>(0.0-90.3)<br>0.031	74.1<br>(0.0-91.2)<br>0.004	43.8<br>(0.0-82.2)<br>0.149	97.0<br>(49.7-99.1)<br>0.000	0.0<br>(0.0-49.5)<br>0.671	N/A	82.9<br>(0.0-96.6)<br>0.016
**WLHIV PI-ART vs HIV-negative women**	Preconception	83.0<br>(0.0, 95,0)<br>0.001	80.4<br>(0.0, 95.6)<br>0.006	45.3<br>(0.0, 89.1)<br>0.176	0.0<br>(0.0, 68.6)<br>0.426	99.1<br>(0.0, 99.8)<br>0.000	N/A		N/A
	Antenatal	77.4<br>(0.0, 95.0)<br>0.012	55.4<br>(0.0, 91.1)<br>0.134	0.0<br>(0.0, 0.0)<br>0.958	18.0<br>(0.0, 83.7)<br>0.269	44.8<br>(0.0, 89.0)<br>0.178			
**WLHIV INSTI-ART vs HIV-negative women**	Antenatal	N/A	N/A			N/A	N/A		N/A

Appendix 4.2: Country Income Status

4.2.1: Subgroup analysis based on High Income Countries and Low- and Middle-Income Countries: Risk Ratios:

	Country Income Status	**Perinatal outcomes**										
		**PTB**	**vPTB**	**sPTB**	**LBW**	**VLBW**	**Term LBW**	**Preterm LBW**	**SGA**	**VSGA**	**Stillbirth**	**NND**
		RR<br>(95%CI)<br>p-value	RR<br>(95%CI)<br>p-value	RR<br>(95%CI)<br>p-value	RR<br>(95%CI)<br>p-value	RR<br>(95%CI)<br>p-value	RR<br>(95%CI)<br>p-value	RR<br>(95%CI)<br>p-value	RR<br>(95%CI)<br>p-value	RR<br>(95%CI)<br>p-value	RR<br>(95%CI)<br>p-value	RR<br>(95%CI)<br>p-value
**WLHIV NNRTI-ART vs HIV-negative women**	High income countries	1.70<br>(0.40, 7.14)<br>0.470	5.85<br>(3.31, 10.33)<br>0.000		3.57<br>(2.85, 4.47)<br>0.000	8.71<br>(5.26, 14.44)<br>0.000			13.47<br>(10.71, 16.94)<br>0.000			
	Low- and Middle-income countries	1.29<br>(1.19, 1.40)<br>0.000	1.32<br>(0.97, 1.78)<br>0.074		1.37<br>(1.23, 1.52)<br>0.000	1.41<br>(0.79, 2.50)<br>0.247	1.83<br>(0.95, 3.53)<br>0.070	1.17<br>(0.64, 2.14)<br>0.607	1.27<br>(1.16, 1.39)<br>0.000	1.48<br>(1.17, 1.87)<br>0.001	0.88<br>(0.34, 2.32)<br>0.799	1.20<br>(0.89, 1.61)<br>0.226
**WLHIV PI-ART vs HIV-negative women**	High income countries	1.91<br>(1.41, 2.58)<br>0.000	2.00<br>(0.41, 9.79)<br>0.392	16.96<br>(1.01, 284.08)<br>0.049	2.98<br>(2.50, 3.55)<br>0.000	4.50<br>(2.75, 7.38)<br>0.000	1.83<br>(0.88, 3.80)<br>0.105		4.65<br>(1.08, 20.06)<br>0.040	1.20<br>(0.29, 4.92)<br>0.800		0.40<br>(0.02, 8.21)<br>0.551
	Low- and Middle-income countries	1.71<br>(1.48, 1.98)<br>0.000	1.83<br>(1.27, 2.67)<br>0.001		1.84<br>(0.77, 4.43)<br>0.172	1.33<br>(0.07, 25.11)<br>0.850			1.63<br>(1.34. 2.00)<br>0.000	2.42<br>(1.87, 3.13)<br>0.000		2.13<br>(1.18, 3.83)<br>0.012
**WLHIV INSTI-ART vs HIV-negative women**	High income countries	1.18<br>(0.76, 1.81)<br>0.463										
	Low- and Middle-income countries	1.17<br>(1.06, 1.30)<br>0.002	1.09<br>(0.86, 1.39)<br>0.464						1.20<br>(1.08, 1.33)<br>0.001	1.15<br>(0.95, 1.39)<br>0.153		0.88<br>(0.89, 1.61)<br>0.226

A RR > 1 indicates increased risk of a perinatal outcome associated with the specified ART class. For example, NNRTI-ART is associated with an increased risk of preterm birth compared to HIV negative women in low- and middle- income countries (RR 1.29 95% CI 1.19, 1.40, p=0.000).

Abbreviations: ART= antiretroviral therapy, HIV= human immunodeficiency virus, PI = protease inhibitor, NNRTI = non-nucleoside reverse transcriptase inhibitor, INSTI = integrase strand transfer inhibitor, LBW= low birthweight, NND= neonatal death, PTB= preterm birth, RR= risk ratio, SGA= small for gestational age, sPTB= spontaneous preterm birth, VLBW= very low birthweight, VPTB= very preterm birth, VSGA= very small for gestational age.

4.2.2: Subgroup analysis based on High Income Countries and Low- and Middle-Income Countries: Number of studies and women analysed

		**Perinatal outcomes**										
		**PTB**	**vPTB**	**sPTB**	**LBW**	**VLBW**	**Term LBW**	**Preterm LBW**	**SGA**	**VSGA**	**Stillbirth**	**NND**
	Country Income Status	Number of studies (Number of women analysed) Events/total for each group	Number of studies (Number of women analysed) Events/total for each group	Number of studies (Number of women analysed) Events/total for each group	Number of studies (Number of women analysed) Events/total for each group	Number of studies (Number of women analysed) Events/total for each group	Number of studies (Number of women analysed) Events/total for each group	Number of studies (Number of women analysed) Events/total for each group	Number of studies (Number of women analysed) Events/total for each group	Number of studies (Number of women analysed) Events/total for each group	Number of studies (Number of women analysed) Events/total for each group	Number of studies (Number of women analysed) Events/total for each group
**WLHIV NNRTI-ART<br>Vs<br>HIV-negative women**	High income countries	2<br>(12470)<br>76/467<br>HIV+<br>928/12003<br>HIV-	1<br>(8977)<br>15/438<br>HIV+<br>50/8539<br>HIV-		1<br>(8977)<br>76/438<br>HIV+<br>415/8539<br>HIV-	1<br>(8977)<br>21/438<br>HIV+<br>47/8539<br>HIV-			1<br>(8977)<br>105/438<br>HIV+<br>152/8539<br>HIV-			
	Low- and Middle-income countries	12<br>(128352)<br>4256/21435<br>HIV+<br>16506/106917<br>HIV-	5<br>(97249)<br>427/10876<br>HIV+<br>3090/86373<br>HIV-		8<br>(31420)<br>1480/10614<br>HIV+<br>2170/20806<br>HIV-	3<br>(3258)<br>36/1765<br>HIV+<br>23/1493<br>HIV-	1<br>(1299)<br>23/614<br>HIV+<br>14/685<br>HIV-	1<br>(1299)<br>21/614<br>HIV+<br>20/685<br>HIV-	11<br>(125728)<br>3023/20209<br>HIV+<br>14311/105519<br>HIV-	5<br>(96341)<br>800/10440<br>HIV+<br>4601/85901<br>HIV-	1<br>(633)<br>6/229<br>HIV+<br>12/404<br>HIV-	4<br>(104305)<br>176/13378<br>HIV+<br>1187/90927<br>HIV-
**WLHIV PI-ART<br>Vs<br>HIV-negative women**	High income countries	6<br>(14,403)<br>224/1,511<br>HIV+<br>1007/12,892<br>HIV-	2<br>(9,767)<br>28/1028<br>HIV+<br>62/8739<br>HIV-	1<br>(104)<br>9/55<br>HIV+<br>0/49<br>HIV-	2<br>(9829)<br>146/1002<br>HIV+<br>432/8827<br>HIV-	1<br>(9467)<br>23/928<br>HIV+<br>47/8539<br>HIV-			4<br>(10,233)<br>263/1157<br>HIV+<br>205/9076<br>HIV-	1<br>(300)<br>3/100<br>HIV+<br>5/200<br>HIV-		1<br>(300)<br>0/100<br>HIV+<br>2/200<br>HIV-
	Low- and Middle-income countries	3<br>(57145)<br>178/653<br>HIV+	2<br>(34841)<br>27/427<br>HIV+		1<br>(307)<br>5/29<br>HIV+	1<br>(307)<br>0/29<br>HIV+	1<br>(380)<br>17/174<br>HIV+		2<br>(34,741)<br>101/427<br>HIV+	1<br>(34536)<br>52/398<br>HIV+		1<br>(34536)<br>11/398<br>HIV+

		9,158/56,492<br>HIV-	1265/34414<br>HIV-		26/278<br>HIV-	3/278<br>HIV-	11/206<br>HIV-		5083/34316<br>HIV-	1843/34138<br>HIV-		444/34138<br>HIV-
**WLHIV INSTI-ART<br><br>Vs<br><br>HIV-negative women**	High income countries	1<br>(3559)<br>18/91<br>HIV+<br>487/3464<br>HIV-										
	Low- and Middle-income countries	1<br>(52375)<br>309/1692<br>HIV+<br>7884/50683<br>HIV-	1<br>(52375)<br>66/1692<br>HIV+<br>1807/50683<br>HIV-						1<br>(51850)<br>297/1678<br>HIV+<br>7419/50172<br>HIV-	1<br>(51850)<br>104/1678<br>HIV+<br>2708/50172<br>HIV-		1<br>(51756)<br>21/1701<br>HIV+<br>21/50055<br>HIV-

4.2.3: Subgroup analysis based on High Income Countries and Low- and Middle-Income Countries: I^2 values:

	Country Income Status	Perinatal outcomes										
		**PTB**	**vPTB**	**sPTB**	**LBW**	**VLBW**	**Term LBW**	**Preterm LBW**	**SGA**	**VSGA**	**Stillbirth**	**NND**
		I^2<br>(95%CI)<br>(p value)	I^2<br>(95%CI)<br>(p value)	I^2<br>(95%CI)<br>(p value)	I^2<br>(95%CI)<br>(p value)	I^2<br>(95%CI)<br>(p value)	I^2<br>(95%CI)<br>(p value)	I^2<br>(95%CI)<br>(p value)	I^2<br>(95%CI)<br>(p value)	I^2<br>(95%CI)<br>(p value)	I^2<br>(95%CI)<br>(p value)	I^2<br>(95%CI)<br>(p value)
**WLHIV NNRTI-ART<br><br>Vs<br><br>HIV-negative women**	High income countries	85.6<br>(0.0, 97.1)<br>0.008	N/A		N/A	N/A			N/A			
	Low- and Middle-income countries	72.8<br>(0.0, 89.0)<br>0.000	71.7<br>(0.0, 92.7)<br>0.007		44.1<br>(0.00, 77.0)<br>0.085	0.0<br>(0.0, 24.4)<br>0.713	N/A	N/A	68.5<br>(0.0, 87.7)<br>0.000	78.2<br>(0.0, 94.5)<br>0.001	N/A	56.8<br>(0.0, 87.1)<br>0.074
**WLHIV PI-ART**	High income countries	64.1<br>(0.0, 87.3)<br>0.016	87.5<br>(0.0, 97.5)<br>0.005	N/A	0.0<br>(0.00, 78.7)<br>0.334	N/A	N/A		96.4<br>(0.0, 99.1)<br>0.000	N/A		N/A

**Vs**<br>**HIV-negative women**	Low- and Middle-income countries	15.4<br>(0.0, 77.2)<br>0.307	0.0<br>(0.0, 0.0)<br>0.993		N/A	N/A	N/A		1.7<br>(0.0, 80.4)<br>0.313	N/A		N/A
**WLHIV INSTI-ART**<br>**Vs**<br>**HIV-negative women**	High income countries	N/A										
	Low- and Middle-income countries	N/A	N/A						N/A	N/A		N/A

Abbreviations: ART= antiretroviral therapy, HIV= human immunodeficiency virus, PI = protease inhibitor, NNRTI = non-nucleoside reverse transcriptase inhibitor, INSTI = integrase strand transfer inhibitor, LBW= low birthweight, NND= neonatal death, PTB= preterm birth, SGA= small for gestational age, sPTB= spontaneous preterm birth, VLBW= very low birthweight, VPTB= very preterm birth, Preterm LBW = Preterm Low birthweight, VSGA= very small for gestational age.

Appendix 4.3: Study Quality: good, average and low quality studies

4.3.1: Subgroup analysis based on study quality: Risk ratios

		**Perinatal outcomes**										
		**PTB**	**VPTB**	**sPTB**	**LBW**	**VLBW**	**Term LBW**	**Preterm LBW**	**SGA**	**VSGA**	**Stillbirth**	**NND**
		RR<br>(95%CI)<br>p-value	RR<br>(95%CI)<br>p-value	RR<br>(95%CI)<br>p-value	RR<br>(95%CI)<br>p-value	RR<br>(95%CI)<br>p-value	RR<br>(95%CI)<br>p-value	RR<br>(95%CI)<br>p-value	RR<br>(95%CI)<br>p-value	RR<br>(95%CI)<br>p-value	RR<br>(95%CI)<br>p-value	RR<br>(95%CI)<br>p-value
**WLHIV NNRTI-ART vs HIV-negative women**	Good quality studies	1.36<br>(0.94, 1.95)<br>0.101	1.29<br>(0.60, 2.77)<br>0.507		1.24<br>(0.89, 1.73)<br>0.214	1.09<br>(0.46, 2.58)<br>0.852			1.46<br>(1.03, 2.06)<br>0.032	1.35<br>(0.67, 2.73)<br>0.404	0.88<br>(0.34, 2.32)<br>0.799	6.18<br>(1.29, 29.48)<br>0.022
	Average quality studies	1.38<br>(1.21, 1.58)<br>0.000	1.85<br>(1.13, 3.01)<br>0.014		1.63<br>(1.25, 2.13)<br>0.000	4.57<br>(1.06, 19.58)<br>0.041	1.83<br>(0.95, 3.53)<br>0.070	1.17<br>(0.64, 2.14)<br>0.607	1.62<br>(1.19, 2.20)<br>0.002	1.48<br>(1.11, 1.96)<br>0.007		1.11<br>(0.93, 1.34)<br>0.258
	Poor quality studies	1.53<br>(1.14, 2.05)<br>0.004	6.02<br>(1.72, 21.05)<br>0.005		2.31<br>(0.65, 8.22)<br>0.196	1.59<br>(0.58, 4.37)<br>0.366			1.35<br>(0.99, 1.85)<br>0.057	1.58<br>(0.79, 3.17)<br>0.193		
**WLHIV PI-ART vs HIV-negative women**	Good quality studies											
	Average quality studies	1.90<br>(1.53-2.36)<br>0.000	2.06<br>(1.01-4.19)<br>0.047	16.96<br>(1.01-284.08)<br>0.049	2.90<br>(2.41-3.50)<br>0.000	4.35<br>(2.68-7.09)<br>0.000			2.92<br>(0.95-9.00)<br>0.062	2.37<br>(1.84,3.05)<br>0.000		1.82<br>(0.71, 4.70)<br>0.212
	Poor quality studies	1.74<br>(1.11-2.71)<br>0.015					1.83<br>(0.88-3.80)<br>0.105					N/A
**WLHIV INSTI-ART vs HIV-negative women**	Good quality studies											
	Average quality studies	1.17<br>(1.06, 1.30)<br>0.002	1.09<br>(0.86, 1.39)<br>0.464						1.20<br>(1.08, 1.33)<br>0.001	1.15<br>(0.95, 1.39)<br>0.153		0.89<br>(0.58, 1.37)<br>0.585

	Poor quality studies											

A RR > 1 indicates increased risk of a perinatal outcome associated with the specified ART class. For example, NNRTI-ART is associated with an increased risk of preterm birth compared to HIV negative women in average quality studies (RR 1.38 95% CI 1.21, 1.58, p=0.000).

Abbreviations: ART=antiretroviral therapy, HIV= human immunodeficiency virus, PI = protease inhibitor, NNRTI = non-nucleoside reverse transcriptase inhibitor, INSTI = integrase strand transfer inhibitor, LBW= low birthweight, NND= neonatal death, PTB= preterm birth, RR= risk ratio, SGA= small for gestational age, sPTB= spontaneous preterm birth, VLBW= very low birthweight, VPTB= very preterm birth, Preterm LBW = Preterm low birthweight, VSGA= very small for gestational age.

4.3.2: Subgroup analysis based on study quality: Number of studies and women analysed

		Perinatal outcomes										
		**PTB**	**VPTB**	**sPTB**	**LBW**	**VLBW**	**Term LBW**	**Preterm LBW**	**SGA**	**VSGA**	**Stillbirth**	**NND**
		Number of studies (number of women analysed) Events/total for each group	Number of studies (Number of women analysed) Events/total for each group	Number of studies (Number of women analysed) Events/total for each group	Number of studies (Number of women analysed) Events/total for each group	Number of studies (Number of women analysed) Events/total for each group	Number of studies (Number of women analysed) Events/total for each group	Number of studies (Number of women analysed) Events/total for each group	Number of studies (Number of women analysed) Events/total for each group	Number of studies (Number of women analysed) Events/total for each group	Number of studies (Number of women analysed) Events/total for each group	Number of studies (Number of women analysed) Events/total for each group
**WLHIV NNRTI-ART vs HIV-negative women**	Good quality studies	1<br>(633)<br>43/229<br>HIV+<br>56/404<br>HIV-	1<br>(633)<br>11/229<br>HIV+<br>15/404<br>HIV-		1<br>(633)<br>47/229<br>HIV+<br>67/404<br>HIV-	1<br>(633)<br>8/229<br>HIV+<br>13/404<br>HIV-			1<br>(633)<br>48/229<br>HIV+<br>58/404<br>HIV-	1<br>(633)<br>13/229<br>HIV+<br>17/404<br>HIV-	1<br>(633)<br>6/229<br>HIV+<br>12/404<br>HIV-	1<br>(633)<br>7/229<br>HIV+<br>2/404<br>HIV-
	Average quality studies	10<br>(136550)<br>3968/19735<br>HIV+<br>17233/116815HIV-	4<br>(104097)<br>418/10459<br>HIV+<br>3122/93638<br>HIV-		6<br>(37965)<br>1404/10038<br>HIV+<br>2435/27927<br>HIV-	2<br>(10209)<br>41/1392<br>HIV+<br>50/8817<br>HIV-	1<br>(1299)<br>23/614<br>HIV+<br>14/685<br>HIV-	1<br>(1299)<br>21/614 HIV+<br>20/685 HIV-	9<br>(132557)<br>1299/19724<br>HIV+<br>14330/112833<br>HIV-	3<br>(94625)<br>765/9630<br>HIV+<br>4572/84995<br>HIV-		3<br>(103672)<br>169/13149<br>HIV+<br>1185/90523<br>HIV-
	Poor quality studies	3<br>(3639)<br>321/1938<br>HIV+<br>145/1701<br>HIV-	1<br>(1496)<br>13/626<br>HIV+<br>3/870<br>HIV-		2<br>(1799)<br>105/785<br>HIV+<br>83/1014<br>HIV-	1<br>(1393)<br>8/582<br>HIV+<br>7/811<br>HIV-			2<br>(1515)<br>81/694<br>HIV+<br>75/821<br>HIV-	1<br>(1083)<br>22/581<br>HIV+<br>12/502<br>HIV-		

**WLHIV PI-ART vs HIV-negative women**	Good quality studies											
	Average quality studies	7<br>(70883)<br>366/1900 HIV+<br>10132/68983 HIV-	4<br>(44608)<br>55/1455 HIV+<br>1327/43153<br>HIV-	1<br>(104)<br>9/55 HIV+<br>0/49 HIV-	3<br>(10,136)<br>151/1031<br>HIV+<br>458/9105<br>HIV-	2<br>(9774)<br>23/957 HIV+<br>50/8817 HIV-			6<br>(45076)<br>364/1584 HIV+<br>5288/43492 HIV-	2<br>(34836)<br>55/498 HIV+<br>1848/34338<br>HIV-		2<br>(34836)<br>11/498 HIV+<br>446/34338<br>HIV-
	Poor quality studies	2<br>(665)<br>36/264 HIV+<br>33/401 HIV-			N/A		1<br>(380)<br>17/174 HIV+<br>11/206<br>HIV-		N/A			N/A
**WLHIV INSTI-ART vs HIV-negative women**	Good quality studies											
	Average quality studies	2<br>(55948)<br>327/1801<br>HIV+<br>8371/54147) HIV-	1<br>(52375)<br>66/1692 HIV+<br>1807/50683<br>HIV-						1<br>(51850)<br>297/1678 HIV+<br>7419/50172<br>HIV-	1<br>(51850)<br>104/1678<br>HIV+<br>2708/50172<br>HIV-		1<br>(51756)<br>21/1701 HIV+<br>21/50055<br>HIV-
	Poor quality studies											

Abbreviations: ART= antiretroviral therapy, HIV= human immunodeficiency virus, PI = protease inhibitor, NNRTI = non-nucleoside reverse transcriptase inhibitor, INSTI = integrase strand transfer inhibitor, LBW= low birthweight, NND= neonatal death, PTB= preterm birth, SGA= small for gestational age, sPTB= spontaneous preterm birth, VLBW= very low birthweight, VPTB= very preterm birth, Preterm LBW = Preterm Low birthweight, VSGA= very small for gestational age.

4.3.3: Subgroup analysis based on study quality: I^2 values

		Perinatal outcomes										
		**PTB**	**VPTB**	**sPTB**	**LBW**	**VLBW**	**Term LBW**	**Preterm LBW**	**SGA**	**VSGA**	**Stillbirth**	**NND**
		I^2 (95%CI) (p value)	I^2 (95%CI) (p value)	I^2 (95%CI) (p value)	I^2 (95%CI) (p value)	I^2 (95%CI) (p value)	I^2 (95%CI) (p value)	I^2 (95%CI) (p value)	I^2 (95%CI) (p value)	I^2 (95%CI) (p value)	I^2 (95%CI) (p value)	I^2 (95%CI) (p value)
**WLHIV NNRTI-ART vs HIV-negative women**	Good quality studies	N/A	N/A		N/A	N/A			N/A	N/A	N/A	N/A
	Average quality studies	90.7 (27.0, 96.6) 0.000	92.0 (0.0, 98.2) 0.000		92.7 (6.7, 97.6) 0.000	80.2 (0.0, 96.1) 0.025	N/A	N/A	98.1 (77.5, 99.4) 0.000	89.0 (0.0, 97.6) 0.000		14.9 (0.0, 77.0) 0.309
	Poor quality studies	56.6 (0.0, 88.3) 0.100	N/A		90.1 (0.0, 98.0) 0.002	N/A			0.0 (0.0, 47.3) 0.539	N/A		
**WLHIV PI-ART vs HIV-negative women**	Good quality studies											
	Average quality studies	72.5 (0.0-89.9) 0.001	73.4 (0.0-92.8) 0.010	N/A	1.7 (0.0, 73.3) 0.362	0.0 (0.0-69.1) 0.422			98.1 (0.0-99.5) 0.000	0.0 (0.0-78.3) 0.338		11.8 (0.0-82.4) 0.287
	Poor quality studies	0.0 (0.0-73.3) 0.388			N/A		N/A		N/A			N/A
**WLHIV INSTI-ART vs HIV-negative women**	Good quality studies											
	Average quality studies	0 (0.0, 0.0) 0.998	0 0.000						0.0 0.000	0.0 0.000		0.00 0.000
	Poor quality studies											

Abbreviations: ART= antiretroviral therapy, HIV= human immunodeficiency virus, PI = protease inhibitor, NNRTI = non-nucleoside reverse transcriptase inhibitor, INSTI = integrase strand transfer inhibitor, LBW= low birthweight, NND= neonatal death, PTB= preterm birth, SGA= small for gestational age, sPTB= spontaneous preterm birth, VLBW= very low birthweight, VPTB= very preterm birth, Preterm LBW = Preterm Low birthweight, VSGA= very small for gestational age.

APPENDIX 4.4: DRUG REGIMEN ANALYSIS

4.4.1: SUBGROUP ANALYSIS OF STUDIES INVOLVING EFAVIRENZ OR NEVIRAPINE CONTAINING NNRTI-BASED ART: RISK RATIOS, NUMBER OF WOMEN ANALYSED, AND I^2 VALUES

		Perinatal outcomes									
		**PTB**	**VPTB**	**LBW**	**VLBW**	**Term LBW**	**Preterm LBW**	**SGA**	**VSGA**	**Stillbirth**	**NND**
		RR<br>(95%CI)<br>p-value	RR<br>(95%CI)<br>p-value	RR<br>(95%CI)<br>p-value	RR<br>(95%CI)<br>p-value	RR<br>(95%CI)<br>p-value	RR<br>(95%CI)<br>p-value	RR<br>(95%CI)<br>p-value	RR<br>(95%CI)<br>p-value	RR<br>(95%CI)<br>p-value	RR<br>(95%CI)<br>p-value
**WLHIV NNRTI-ART vs HIV-negative women**	EFV	1.28<br>(1.18, 1.39)<br>0.000	1.06<br>(0.93, 1.22)<br>0.381	1.31<br>(1.21, 1.42)<br>0.000	1.32<br>(0.65, 2.65)<br>0.442	1.83<br>(0.95, 3.53)<br>0.070	1.17<br>(0.64, 2.14)<br>0.607	1.20<br>(1.15, 1.26)<br>0.000	1.29<br>(1.19, 1.41)<br>0.000	0.88<br>(0.34, 2.32)<br>0.799	1.05<br>(0.87, 1.23)<br>0.626
	NVP	1.26<br>(1.04, 1.53)<br>0.017	1.51<br>(1.26, 1.82)<br>0.000	1.45<br>(1.30, 1.62)<br>0.000				1.65<br>(1.55, 1.76)<br>0.000	2.28<br>(2.01, 2.57)<br>0.000		1.48<br>(1.08, 2.04)<br>0.015

		Perinatal outcomes									
		**PTB**	**VPTB**	**LBW**	**VLBW**	**Term LBW**	**Preterm LBW**	**SGA**	**VSGA**	**Stillbirth**	**NND**
		Number of studies (number of women analysed) Events/total for each group	Number of studies (Number of women analysed) Events/total for each group	Number of studies (Number of women analysed) Events/total for each group	Number of studies (Number of women analysed) Events/total for each group	Number of studies (Number of women analysed) Events/total for each group	Number of studies (Number of women analysed) Events/total for each group	Number of studies (Number of women analysed) Events/total for each group	Number of studies (Number of women analysed) Events/total for each group	Number of studies (Number of women analysed) Events/total for each group	Number of studies (Number of women analysed) Events/total for each group
**WLHIV NNRTI-ART vs HIV-negative women**	EFV	10<br>(117029)<br>3299/16593 HIV+<br>10844/100436 HIV-	4<br>(93628)<br>295/8125 HIV+<br>3087/8125 HIV-	5<br>(21139)<br>1041/7526 HIV+<br>1463/13613 HIV-	2<br>(2177)<br>32/1495 HIV+<br>16/666 HIV-	1<br>(1299)<br>23/614 HIV+<br>14/685 HIV-	1<br>(1299)<br>21/614 HIV+<br>20/685 HIV-	10<br>(115121)<br>2181/15781 HIV+<br>13721/99340 HIV-	5<br>(94216)<br>HIV+<br>4601/85901 HIV-	1<br>(633)<br>6/229 HIV+<br>12/404 HIV-	4<br>(102180)<br>135/11253 HIV+<br>1187/90927 HIV-
	NVP	3<br>(50661)<br>833/4428 HIV+	1<br>(36263)<br>119/2125 HIV+	3<br>(14804)<br>371/2506 HIV+				3<br>(50661)<br>842/4428 HIV+	1<br>(36263)<br>261/2125 HIV+		1<br>(36263)<br>41/2125 HIV+<br>444/34138 HIV-

		6989/46233 HIV-	1263/34138 HIV-	1283/12298 HIV-				6071/46233 HIV-	1843/34138 HIV-		

		**Perinatal outcomes**									
		**PTB**	**VPTB**	**LBW**	**VLBW**	**Term LBW**	**Preterm LBW**	**SGA**	**VSGA**	**Stillbirth**	**NND**
		I^2 (95%CI) (p value)	I^2 (95%CI) (p value)	I^2 (95%CI) (p value)	I^2 (95%CI) (p value)	I^2 (95%CI) (p value)	I^2 (95%CI) (p value)	I^2 (95%CI) (p value)	I^2 (95%CI) (p value)	I^2 (95%CI) (p value)	I^2 (95%CI) (p value)
**WLHIV NNRTI-ART vs HIV-negative women**	EFV	63.3 (0.0, 85.2) 0.004	8.7 (0.0, 70.8) 0.350	0.0 (0.0, 64.1) 0.719	0.0 (0.0, 0.0) 80.1	N/A	N/A	39.5 (0.0, 69.8) 0.094	0.0 (0.0, 64.1) 0.964	N/A	60.4 (0.0, 84.7) 0.055
	NVP	85.7 (0.0, 96.3) 0.001	N/A	80.1 (0.0, 91.8) 0.007				88.4 (55.8, 94.4) 0.000	N/A		N/A

Abbreviations: ART= antiretroviral therapy, EFV = Efavirenz, NVP = Nevirapine, HIV= human immunodeficiency virus, PI = protease inhibitor, NNRTI = non-nucleoside reverse transcriptase inhibitor, NRTI = nucleoside reverse transcriptase inhibitor, INSTI = integrase strand transfer inhibitor, LBW= low birthweight, NND= neonatal death, PTB= preterm birth, SGA= small for gestational age, sPTB= spontaneous preterm birth, VPTB= very preterm birth, VSGA= very small for gestational age.

APPENDIX 5: SENSITIVITY ANALYSES

Adjusting for confounders in individual studies comparing women living with HIV receiving PI, NNRTI and INSTI-based ART to HIV negative women for preterm birth, very preterm birth, low birthweight, small for gestational age, very small for gestational age, stillbirth and neonatal death.

Study	ART exposure	Perinatal outcome															
		PTB		VPTB		LBW		VLBW		SGA		VSGA		Stillbirth		NND	
		OR/RR (95% CI)	aOR/aRR (95% CI)	OR/RR (95% CI)	aOR/aRR (95% CI)	OR/RR (95% CI)	aOR/aRR (95% CI)	OR/RR (95% CI)	aOR/aRR (95% CI)	OR/RR (95% CI)	aOR/aRR (95% CI)	OR/RR (95% CI)	aOR/aRR (95% CI)	OR/RR (95% CI)	aOR/aRR (95% CI)	OR/RR (95% CI)	aOR/aRR (95% CI)
**Dadabhai et al (2019)**	NNRTI	1.13 (0.79, 1.62)	1.10 (0.73, 1.65)			1.48 (0.93, 2.34)	1.62 (0.97, 2.71)			0.92 (0.69, 1.22)	1.02 (0.75, 1.41)						
**Gagnon et al (2016)**	PI	2.1 (1.0, 4.6)	0.8 (0.2, 2.8)			2.3 (0.9, 5.9)	1.0 (0.2, 4.9)			2.9 (1.4, 6.2)	1.5 (0.4, 5.1)						
**Malaba et al (2017)**	NNRTI	1.96 (1.34, 2.87)	1.97 (1.28, 3.04)			1.65 (1.06, 2.57)	1.51 (0.91, 2.48)			0.81 (0.53, 1.24)	0.96 (0.61, 1.52)						
	PI	3.54 (1.52, 8.21)	4.46 (1.55, 12.83)			2.02 (0.71, 5.74)	2.39 (0.67, 8.57)			0.92 (0.26, 3.22)	1.01 (0.26, 3.92)						
**Malaba et al (2018)**	NNRTI	1.77 (1.21, 2.62)	1.98 (1.12, 3.53)														
**Santosa et al (2019)**	NNRTI	1.38 (0.89, 2.15)	1.40 (0.86, 2.26)	1.26 (0.57, 2.80)	1.26 (0.53, 3.02)	1.26 (0.83, 1.92)	1.15 (0.73, 1.82)	1.06 (0.43, 2.59)	1.14 (0.43, 3.04)	1.55 (1.01, 2.37)	1.45 (0.91, 2.33)	1.33 (0.63, 2.81)	1.50 (0.66, 3.44)	0.88 (0.33, 2.37)	0.77 (0.24, 2.41)	6.15 (1.27, 29.88)	7.82 (1.32, 46.42)
**Zash et al (2017)***	NNRTI	1.41 (1.33, 1.50)	1.39 (1.33, 1.46)	1.29 (1.12, 1.49)	1.42 (1.29, 1.57)					1.46 (1.37, 1.55)	1.47 (1.40, 1.54)	1.76 (1.59, 1.95)	1.63 (1.51, 1.77)			1.17 (0.91, 1.50)	1.22 (1.03, 1.45)
**Zash et al (2018)***	NNRTI	1·18 (1·12, 1·25)	1.18 (1.12, 1.25)	1·01 (0·88, 1.16)	1.01 (0.88, 1.16)					1·23 (1·17, 1.31)	1.30 (1.23, 1.38)	1.21 (1.09, 1·34)	1.28 (1.16, 1.42)			1·10 (0·92,1·30)	1·08 (0·91, 1·29)

HIV negative group used as reference in all cases. Studies marked with * indicates RR/aRR reported, all other studies reporting OR/aOR.

Abbreviations: ART= antiretroviral therapy, HIV= human immunodeficiency virus, PI = protease inhibitor, NNRTI = non-nucleoside reverse transcriptase inhibitor, NRTI = nucleoside reverse transcriptase inhibitor, LBW= low birthweight, NND= neonatal death, PTB= preterm birth, SGA= small for gestational age, sPTB= spontaneous preterm birth, VLBW= very low birthweight, VPTB= very preterm birth, VSGA= very small for gestational age.

Appendix 6: Funnel plots

Figure 6.1: Preterm birth in women living with HIV receiving NNRTI-based ART vs HIV negative women

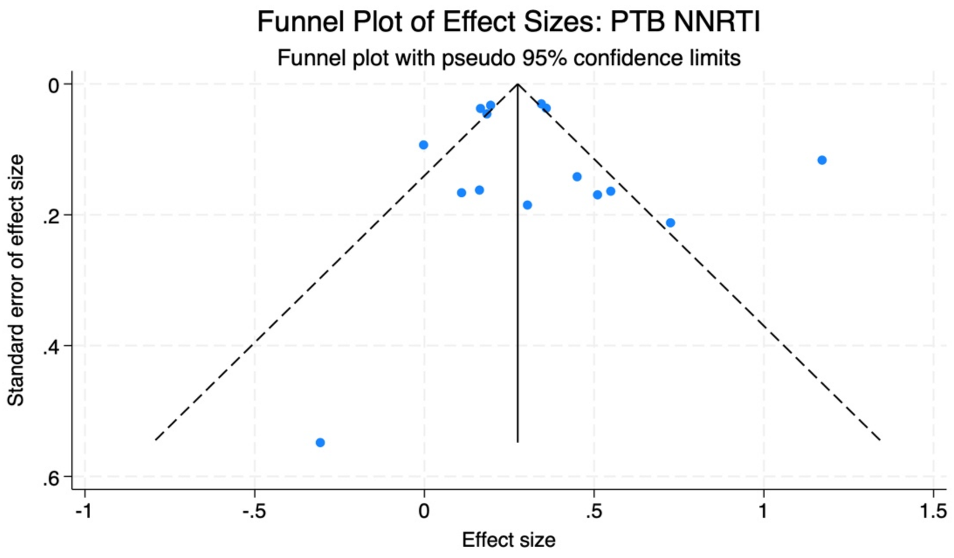

Figure 6.2: Very preterm birth in women living with HIV receiving NNRTI-based ART vs HIV negative women

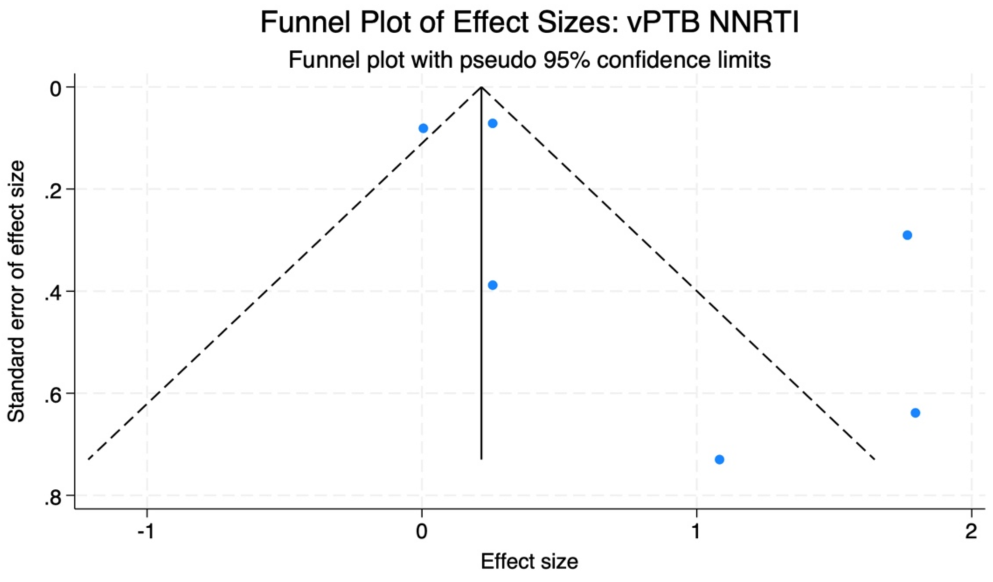

FIGURE 6.3: LOW BIRTHWEIGHT IN WOMEN LIVING WITH HIV RECEIVING NNRTI-BASED ART VS HIV NEGATIVE WOMEN

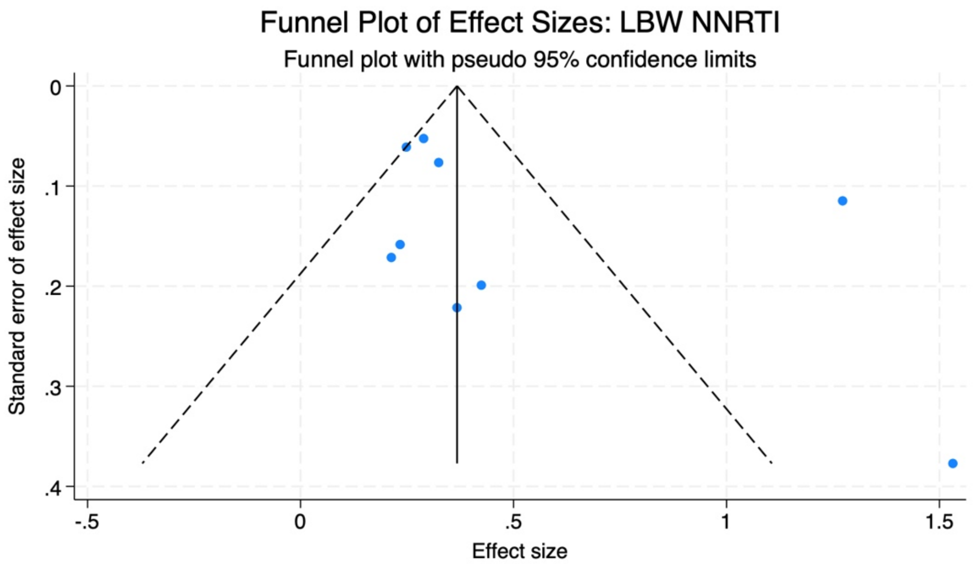

FIGURE 6.4: VERY LOW BIRTHWEIGHT IN WOMEN LIVING WITH HIV RECEIVING NNRTI-BASED ART VS HIV NEGATIVE WOMEN

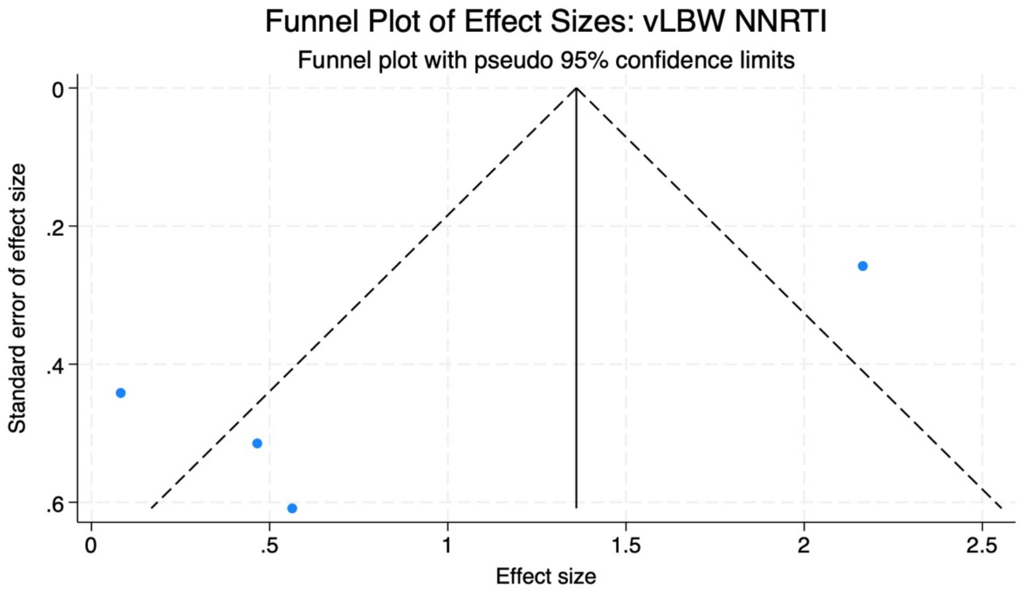

FIGURE 6.5: SMALL FOR GESTATIONAL AGE IN WOMEN LIVING WITH HIV RECEIVING NNRTI-BASED ART VS HIV NEGATIVE WOMEN

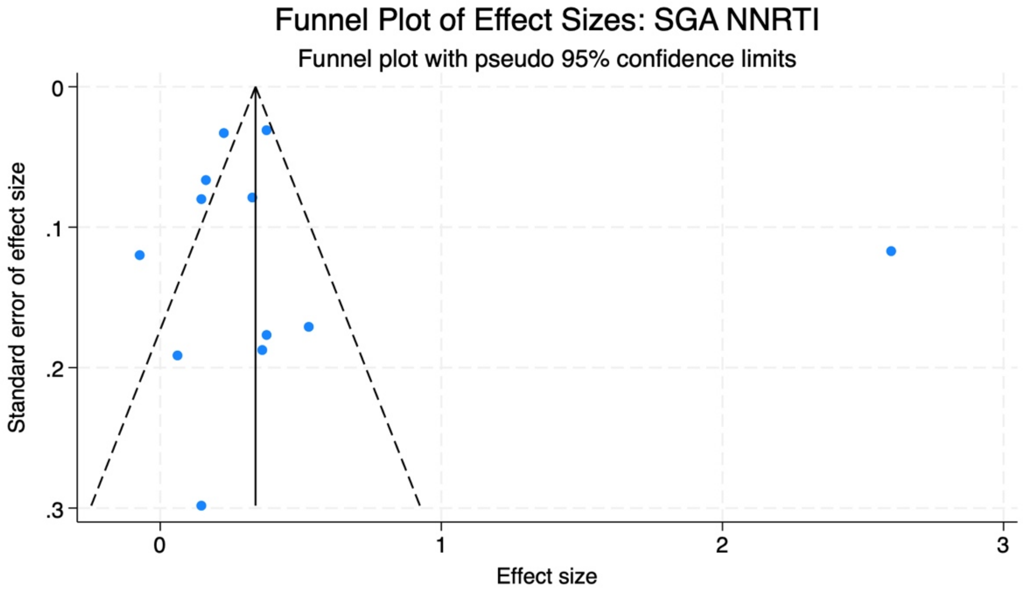

FIGURE 6.6: VERY SMALL FOR GESTATIONAL AGE IN WOMEN LIVING WITH HIV RECEIVING NNRTI-BASED ART VS HIV NEGATIVE WOMEN

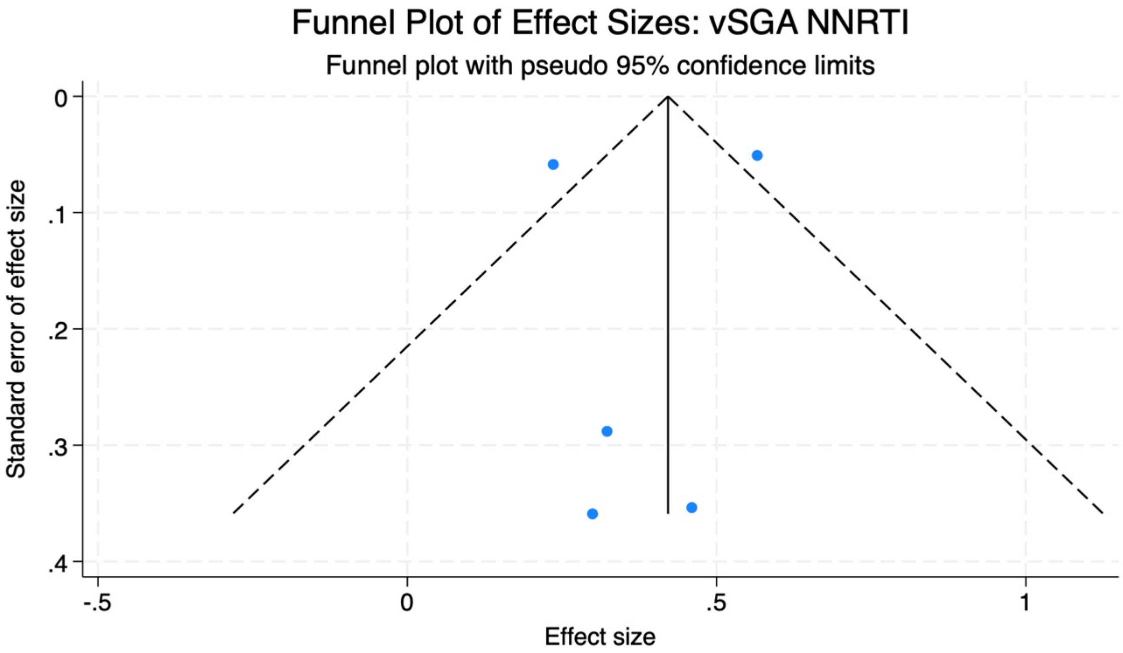

FIGURE 6.7: NEONATAL DEATH IN WOMEN LIVING WITH HIV RECEIVING NNRTI-BASED ART VS HIV NEGATIVE WOMEN

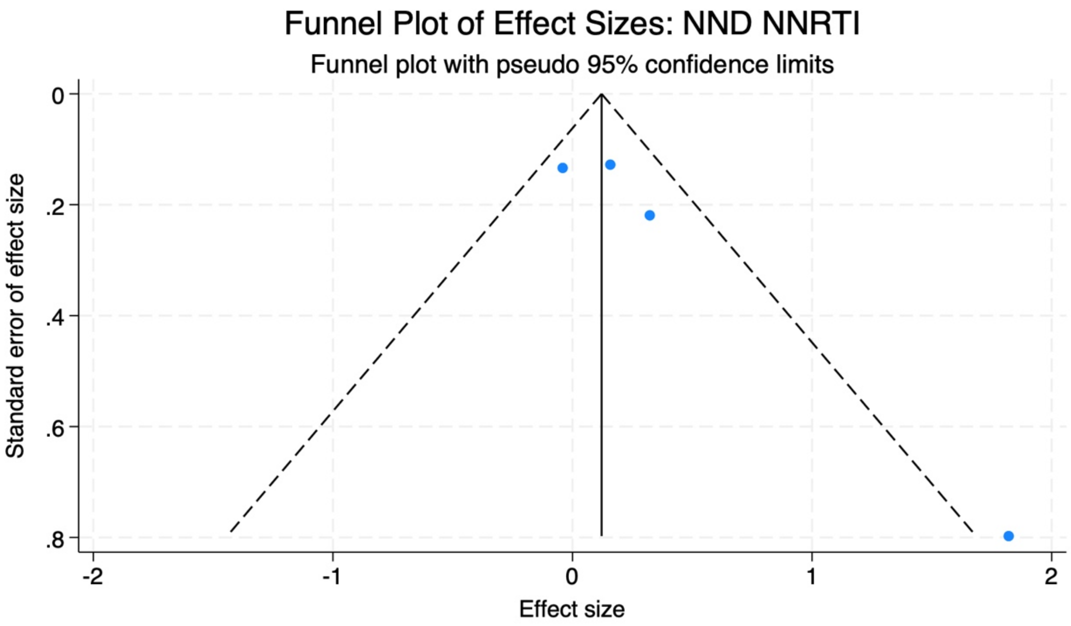

FIGURE 6.8: PRETERM BIRTH IN WOMEN LIVING WITH HIV RECEIVING PI-BASED ART VS HIV NEGATIVE WOMEN

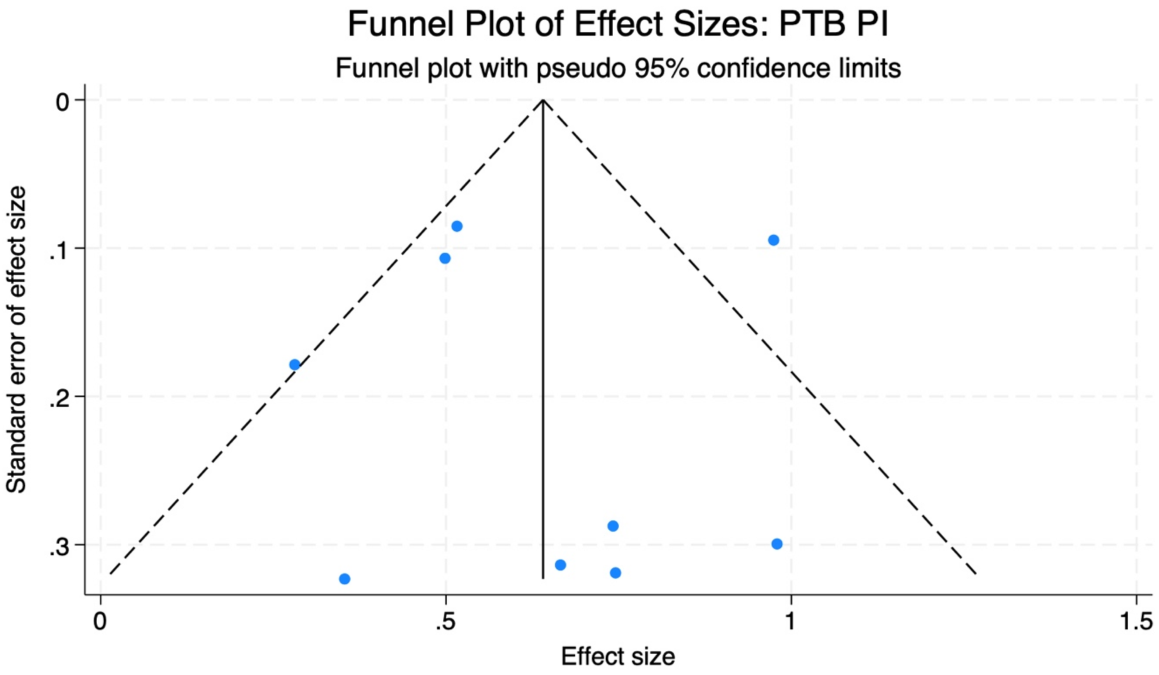

FIGURE 6.9: VERY PRETERM BIRTH IN WOMEN LIVING WITH HIV RECEIVING PI-BASED CART VS HIV NEGATIVE WOMEN

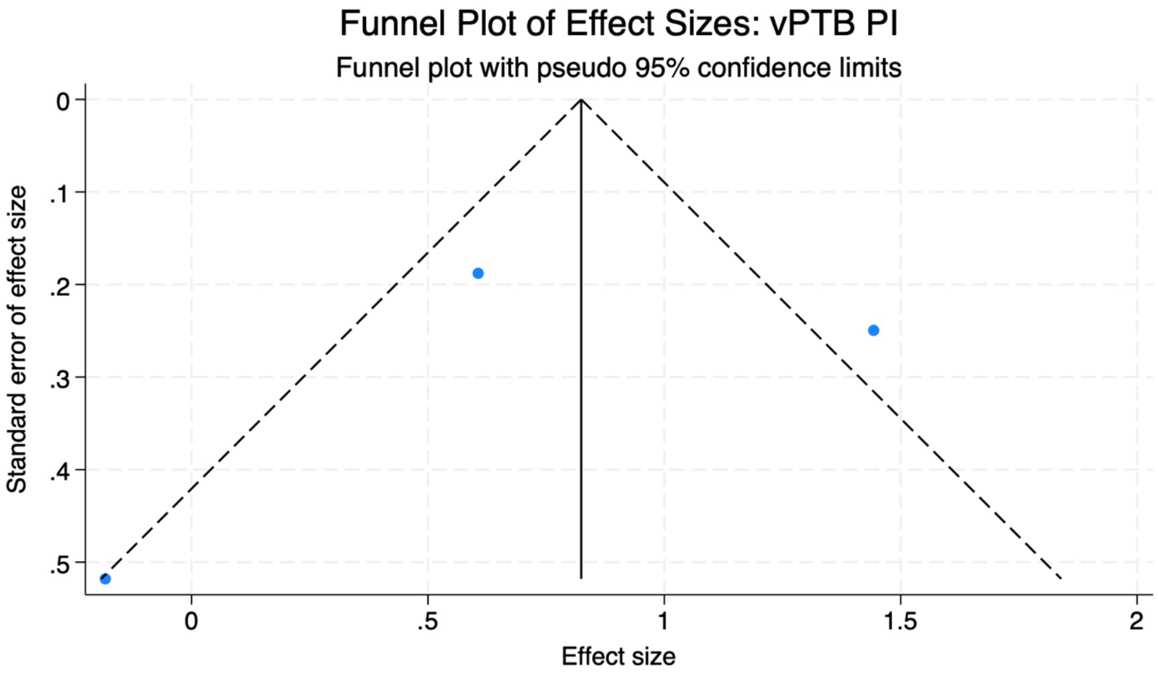

FIGURE 6.10 LOW BIRTHWEIGHT IN WOMEN LIVING WITH HIV RECEIVING PI-BASED ART VS HIV NEGATIVE WOMEN

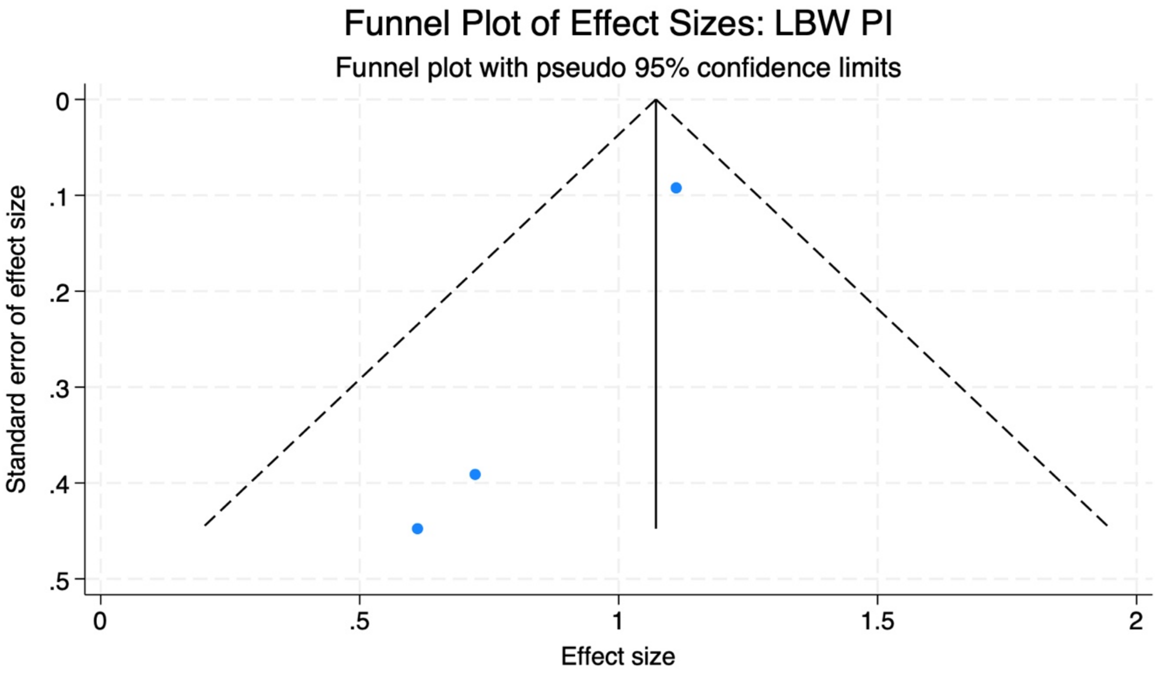

FIGURE 6.11: VERY LOW BIRTHWEIGHT IN WOMEN LIVING WITH HIV RECEIVING PI-BASED ART VS HIV NEGATIVE WOMEN

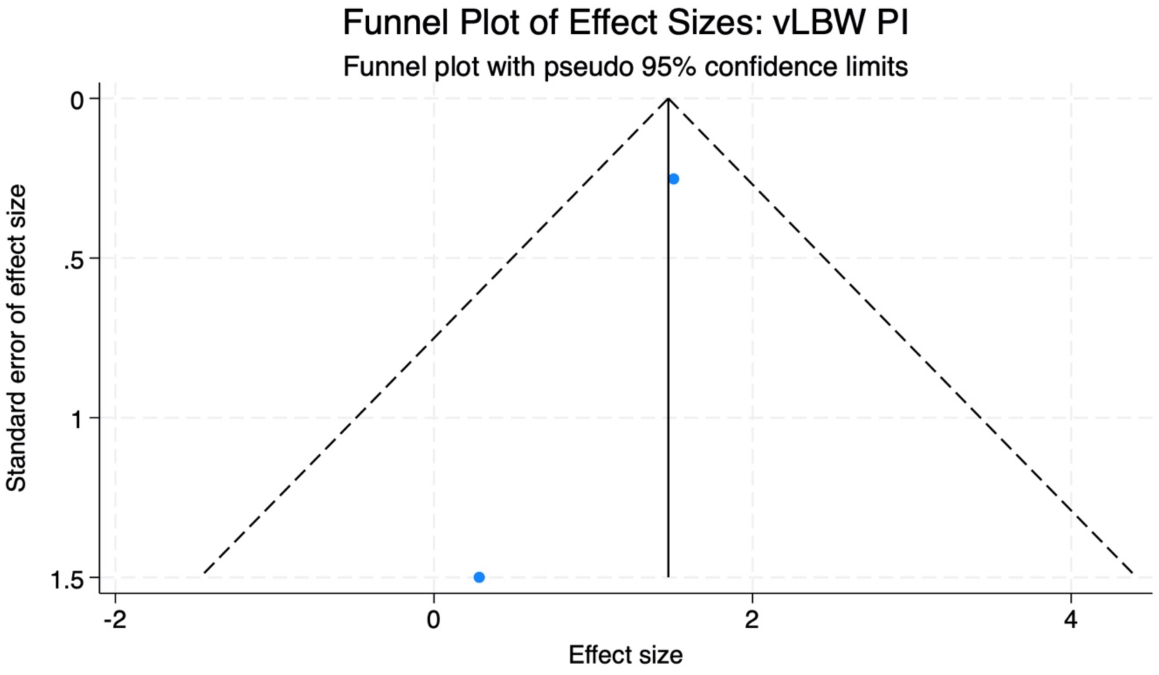

Figure 6.12: Small for gestational age in women living with HIV receiving PI-based ART vs HIV negative women

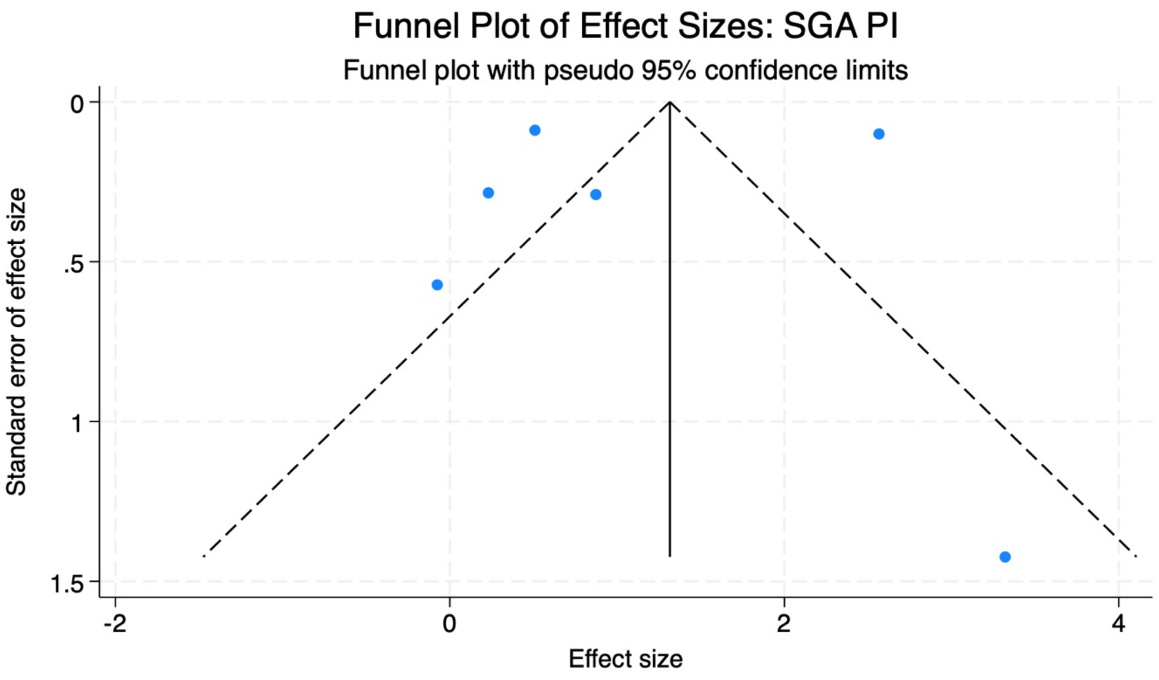

Figure 6.13: very small for gestational age in women living with HIV receiving PI-based ART vs HIV

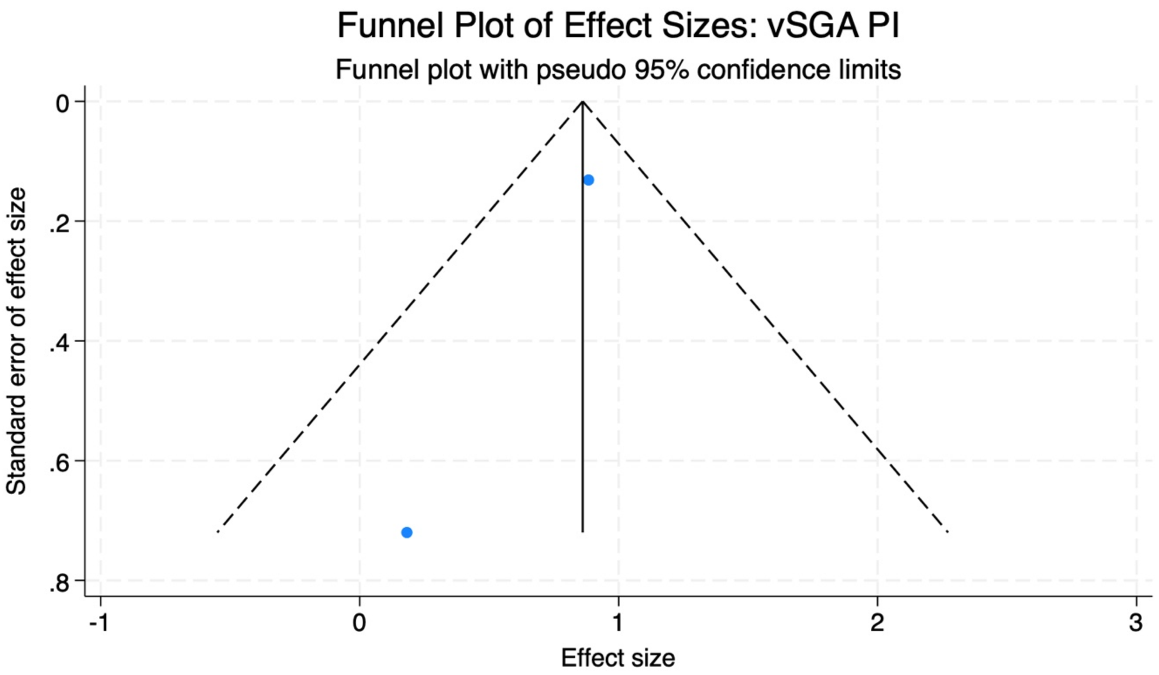

FIGURE 6.14: NEONATAL DEATH IN WOMEN LIVING WITH HIV RECEIVING PI-BASED ART VS HIV NEGATIVE

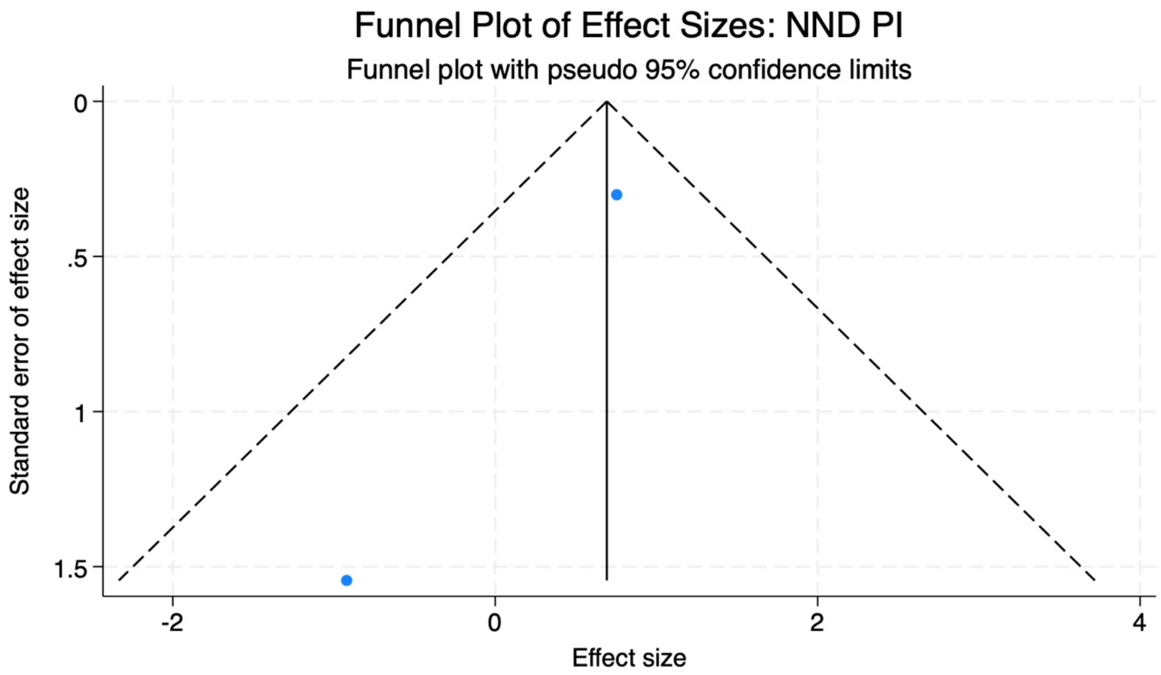

FIGURE 6.15: PTB IN WOMEN LIVING WITH HIV RECEIVING INSTI-BASED ART VS HIV NEGATIVE

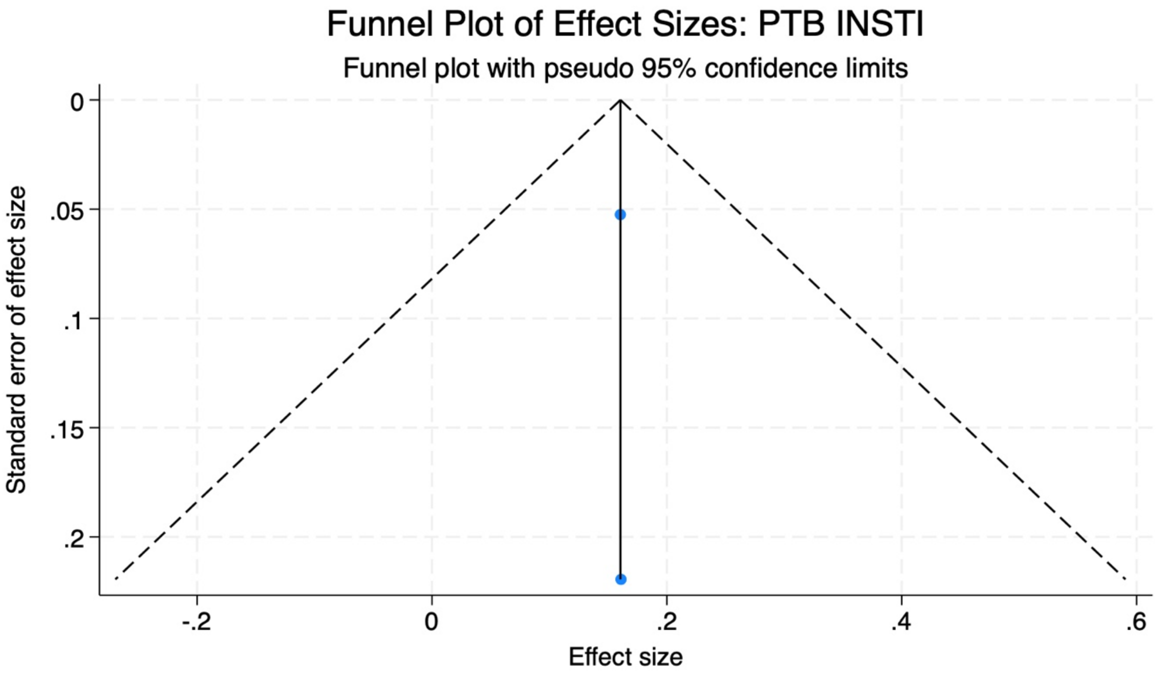

Supplement: Supplemental Digital Content [file aids-39-162-s001.pdf]
